# Supplementary material for: Adherence to physical rehabilitation delivered via tele-rehabilitation for people with multiple sclerosis: a scoping review protocol
Source: BMJ Open. 2023 Mar 8;13(3):e062548. doi: 10.1136/bmjopen-2022-062548 (PMC10008230; doi:10.1136/bmjopen-2022-062548)
Supplement: Supplementary data [file bmjopen-2022-062548supp001.pdf]

Supplementary file

Medline (Ovid):

1 Multiple sclerosis.ti,ab. 85317
2 demyelinat*.ti,ab. 34867
3 multiple sclerosis/ or demyelinating diseases/ 70724
4 1 or 2 or 3 118843
5 (tele* or virtual* or digital* or video or remote or web based or technolog* or online or mobile app* or software or phone or internet).ti,ab. 1620083
6 (exercise* or rehabilitat* or physiotherap* or physical therap*).ti,ab. 541966
7 (physical* adj activ*).ti,ab. 142680
8 6 or 7 649188
9 5 and 8 56897
10 telecommunications/ or telemedicine/ or remote consultation/ or telerehabilitation/ or telephone/ or videoconferencing/ or wireless technology/ 61250
11 Information Technology/ 717
12 internet/ or internet-based intervention/ 80763
13 Mobile Applications/ 10527
14 9 or 10 or 11 or 12 or 13 195458
15 (exercise* or rehabilitat* or physiotherap* or physical therap*).ti,ab. 541966
16 (physical* adj activ*).ti,ab. 142680
17 exp Physical Therapy Modalities/ 173345
18 exp Rehabilitation/ 343384
19 exp Physical Therapists/2853
20 exp Exercise/ 236155
21 15 or 16 or 17 or 18 or 19 or 20 953695
22 4 and 14 and 21696
23 limit 22 to yr="1998 -Current" 690

Draft search strategies:

Embase (Ovid)

1 Multiple Sclerosis.ti,ab. 134911
2 demyelinat*.ti,ab. 54974
3 multiple sclerosis/ or demyelinating disease/ 159457
4 1 or 2 or 3 199828
5 (tele* or virtual* or digital* or video or remote or web based or technolog* or online or mobile app* or software or phone or internet).ti,ab. 2217979
6 (exercise* or rehabilitat* or physiotherap* or physical therap*).ti,ab. 749164
7 (physical* adj activ*).ti,ab. 195167
8 6 or 7 892244
9 5 and 8 87670
10 telecare/ or telecommunication/ or teleconference/ or telehealth/ or telemedicine/ or teleconsultation/ or telemonitoring/ or telerehabilitation/ or video consultation/ 100269
11 telephone interview/ or telephone/ 60517
12 videoconferencing/ 7864
13 wireless communication/ 7098
14 information technology/ 12885
15 internet/ or web-based intervention/ 122028
16 mobile application/ or mobile health application/ 22434
17 9 or 10 or 11 or 12 or 13 or 14 or 15 or 16 376530
18 (exercise* or rehabilitat* or physiotherap* or physical therap*).ti,ab. 749164
19 (physical* adj activ*).ti,ab. 195167
20 exp physiotherapy/ 102325
21 exp rehabilitation/ 462483
22 exp physiotherapist/ 26174
23 exp exercise/ 407525
24 exp physical activity/ 511247
25 18 or 19 or 20 or 21 or 22 or 23 or 24 1599769
26 4 and 17 and 251453
27 limit 26 to yr="1998 -Current" 1445

HMIC Health Management Information Consortium (Ovid)

1 Multiple Sclerosis.ti,ab. 317
2 demyelinat*.ti,ab. 2
3 exp Multiple sclerosis/ 290
4 1 or 2 or 3 391
5 (tele* or virtual* or digital* or video or remote or web based or technolog* or online or mobile app* or software or phone or internet).ti,ab. 21838
6 (exercise* or rehabilitat* or physiotherap* or physical therap*).ti,ab. 9345
7 (physical* adj activ*).ti,ab. 2529
8 6 or 7 11439
9 5 and 8 921
10 telehealth/ or telemedicine/ or telecare/ 2220
11 Telephone/ or Telephone interviewing/ or Telephone advice/ 267
12 information technology/ 5014
13 Internet/ 1352
14 9 or 10 or 11 or 12 or 13 8984
15 (exercise* or rehabilitat* or physiotherap* or physical therap*).ti,ab. 9345
16 (physical* adj activ*).ti,ab. 2529
17 exp exercise/ or exp physical activity/ or exp exercise therapy/ 2964
18 exp Rehabilitation/ 2116
19 exp Physiotherapy/ 910

20	exp Physiotherapists/	363
21	15 or 16 or 17 or 18 or 19 or 20	13304
22	4 and 14 and 214	
23	limit 22 to yr="1998 -Current"	4

Proquest thesis and dissertations

Set	Search	Results
S7	S1 AND S6 AND S4	328
S6	S2 OR S3	51,057
S4	(TI, AB (exercise*)) OR (TI,AB (rehabilitat*)) AND (TI,AB (physiotherap*)) AND (TI,AB (physical therap*)) AND (TI,AB (physical* NEAR/1 activ*))Limits applied	7,641
S3	(TI, AB (phone)) OR (TI,AB (internet))Limits applied	8,963
S2	(TI, AB (tele*)) OR (TI,AB (virtual)) OR (TI,AB (digital)) OR (TI,AB (video)) OR (TI,AB (remote)) OR (TI,AB ("web based")) OR (TI,AB (technolog*)) OR (TI,AB (online)) OR (TI,AB ("mobile app*")) OR (TI,AB (software))Limits applied	52,313
S1	(TI,AB (Multiple sclerosis)) OR (TI,AB (demyelinat*))Limits applied	2,074

Limits applied = search dates 1998 – current

All searches performed on 14 databases

CINAHL

S20	PY 1998-current	107
S19	S4 AND S15 AND S18	408
S18	S16 OR S17	565,738
S17	(MH "Exercise+") OR (MH "Rehabilitation+") OR (MH "Home Rehabilitation+") OR (MH "Physical Therapy+") OR (MH "Physical Therapists")	425,188
S16	TI exercise* OR AB exercise* OR TI rehabilitat* OR AB rehabilitat* OR TI physiotherap* OR AB physiotherap* OR TI "physicial therap*" OR AB "physical therap*" OR TI "physical* N activ*" OR AB "physical* N activ*"	269,339
S15	S9 OR S10 OR S11 OR S12 OR S13 OR S14	159,431
S14	(MH "Mobile Applications")	11,645
S13	(MH "Information Technology")	15,291
S12	(MH "Wireless Communications") OR (MH "Internet") OR (MH "Telephone") OR (MH "Internet-Based Intervention")	84,100
S11	(MH "Videoconferencing") OR (MH "Teleconferencing")	4,983
S10	(MH "Telerehabilitation") OR (MH "Telecommunications") OR (MH "Telehealth") OR (MH "Telemedicine") OR (MH "Remote Consultation")	33,195
S9	S7 AND S8	25,827
S8	S5 OR S6	521,765
S7	TI exercise* OR AB exercise* OR TI rehabilitat* OR AB rehabilitat* OR TI physiotherap* OR AB physiotherap* OR TI "physical therap*" OR AB "physical therap*" OR TI "physical* N activ*" OR AB "physical* N activ*"	273,823
S6	TI technolog* OR AB technolog* OR TI online OR AB online OR TI "mobile app*" OR AB "mobile app*" OR TI software OR AB software OR TI phone OR AB phone OR TI internet OR AB internet	369,239
S5	TI tele* OR AB tele* OR TI virtual* OR AB virtual* OR TI digital* OR AB digital* OR TI video OR AB video OR TI remote OR AB remote OR TI "web based" OR AB "web based"	232,047
S4	S1 OR S2 OR S3	30,771
S3	TI "multiple sclerosis" OR AB "multiple sclerosis" OR TI demyelinat* OR AB demyelinat*	23,546
S2	(MH "Demyelinating Diseases")	2,309
S1	(MH "Multiple Sclerosis")	22,249

Pedro:

Abstract & Title: "multiple sclerosis" tele*
Subdiscipline: neurology
Match all search terms (AND)

World Health Organisation International Clinical Trials Registry Platform portal:

- Multiple sclerosis AND tele* OR remote OR digital OR online OR phone OR internet OR virtual OR web OR software OR technolog* OR mobile OR video OR mobile
- Multiple sclerosis AND exercise* or rehabilitat* or physiotherap* or "physical therap*" or "physical activ*"

US National Library of Medicine Registry of Clinical Trials:

(telerehabilitation OR telemedicine OR telecare OR phone OR digital OR remote OR online OR mobile OR software OR video OR web OR techolog* OR internet) AND (exercise OR rehab* OR "physical activ*" OR "physical therapist") | Multiple Sclerosis

Cochrane central register of controlled trials Date Run: 08/12/2022 15:05:37

ID	Search	Hits
#1	MeSH descriptor: [Multiple Sclerosis] explode all trees	4016
#2	"multiple sclerosis":ti,ab	10983
#3	demyelinat*:ti,ab	1142
#4	#1 or #2 or #3	11891
#5	[mh telerehabilitation] or [mh telecommunications] or [mh telemedicine] or [mh "remote consultation"] or [mh telephone] or [mh "video conferencing"] or [mh "wireless technology"] or [mh internet] or [mh "information technology"] or [mh "mobile applications"]	12743
#6	(tele* or virtual* or video or remote* or "web based" or technolog* or online or "mobile app*" or software or phone or internet):ti,ab	137796
#7	(exercise* or rehabilitat* or physiotherap* or "physical therap*"):ti,ab	146032
#8	(physical* NEXT activ*):ti,ab	36900
#9	#7 or #8	166485
#10	#9 and #6	22937
#11	#10 or #5	33893
#12	[mh rehabilitation] or [mh exercise] or [mh "physical therapy modalities"] or [mh "physical therapy speciality"] or [mh "physical therapists"]	62360
#13	#12 or #9	191391
#14	#13 and #11 and #4 with Cochrane Library publication date from Jan 1998 to present	511

Websites:

The names of websites to be searched alongside search terms are provided below. On each website, authors will review the first three pages of search results. If there is no search function within a website, authors will look under relevant sections on that website, such as ‘publications’ or ‘conferences’.

Websites to be searched:
MS Society, MS Trust, Chartered Society of Physiotherapy, UK Society of Behavioural Medicine, International Society of Behavioural Medicine, Open Grey, National MS Society, Rehabilitation in MS (RIMS), European Committee for Treatment and Research in MS.

Search terms:
Tele, virtual, multiple sclerosis, exercise
