# Supplementary material for: Adherence to physical rehabilitation delivered via tele-rehabilitation for people with multiple sclerosis: a scoping review protocol
Source: BMJ Open. 2023 Mar 8;13(3):e062548. doi: 10.1136/bmjopen-2022-062548 (PMC10008230; doi:10.1136/bmjopen-2022-062548)
Supplement: Author's manuscript [file bmjopen-2022-062548.draft_revisions.pdf]

BMJ Open is committed to open peer review. As part of this commitment we make the peer review history of every article we publish publicly available.

When an article is published we post the peer reviewers' comments and the authors' responses online. We also post the versions of the paper that were used during peer review. These are the versions that the peer review comments apply to.

The versions of the paper that follow are the versions that were submitted during the peer review process. They are not the versions of record or the final published versions. They should not be cited or distributed as the published version of this manuscript.

BMJ Open is an open access journal and the full, final, typeset and author-corrected version of record of the manuscript is available on our site with no access controls, subscription charges or pay-per-view fees (http://bmjopen.bmj.com).

If you have any questions on BMJ Open's open peer review process please email info.bmjopen@bmj.com

BMJ Open

Adherence to physical rehabilitation delivered via tele-rehabilitation for people with Multiple Sclerosis: a scoping review protocol

Journal:	*BMJ Open*
Manuscript ID	bmjopen-2022-062548
Article Type:	Protocol
Date Submitted by the Author:	28-Mar-2022
Complete List of Authors:	Goldsmith, Geraldine; University of Exeter<br>Bollen, Jessica; PenCLAHRC University of Exeter Medical School,<br>Salmon , Victoria ; University of Exeter,<br>Freeman, Jennifer; Peninsula Allied Hlth Ctr, Faculty of Health and Human Sciences<br>Dean, Sarah; PenCLAHRC University of Exeter Medical School,
Keywords:	Multiple sclerosis < NEUROLOGY, Telemedicine < BIOTECHNOLOGY & BIOINFORMATICS, REHABILITATION MEDICINE

SCHOLARONE™
Manuscripts

Adherence to physical rehabilitation delivered via tele-rehabilitation for people with Multiple Sclerosis: a scoping review protocol

Corresponding author:
Geraldine Goldsmith
Exeter Community Neuro-rehabilitation Team, Poltimore Centre, Exeter Community Hospital, Hospital Lane, Exeter, EX1 3RB
g.goldsmith@exeter.ac.uk

Co-authors:

Dr Jessica Bollen
College of Medicine and Health
University of Exeter
Exeter, UK

Dr Victoria Salmon
College of Medicine and Health
University of Exeter
Exeter, UK

Professor Jennifer Freeman
Faculty of Health, School of Health Professions
University of Plymouth
Plymouth, UK

Professor Sarah Dean
College of Medicine and Health
University of Exeter
Exeter, UK

Word count: 3031

For peer review only

For peer review only - http://bmjopen.bmj.com/site/about/guidelines.xhtml

ABSTRACT:

Introduction: Using tele-rehabilitation methods to deliver exercise, physical activity (PA) and behaviour change interventions for people with Multiple Sclerosis (pwMS) has increased in recent years, especially since the SARS-CoV-2 pandemic. This scoping review aims to provide an overview of the literature regarding adherence to therapeutic exercise and PA delivered via tele-rehabilitation for pwMS.

Methods and analysis: Frameworks described by Arksey and O'Malley (2005) and Levac *et al* (2010) underpin the methods. The following databases will be searched from 1998 to present day: Medline (OvidSP), Embase (OvidSP), Cinahl (EBSCOhost), The Health Management Information Consortium Database, ProQuest Dissertations and Theses Global, Pedro, the Cochrane central register of controlled trials, US National Library of Medicine Registry of Clinical Trials, World Health Organisation International Clinical Trials Registry Platform portal, The Cochrane Database of Systematic Reviews, and Epistemonikos. To identify papers not included in databases, conference websites will be searched. With the exception of study protocols, papers of any study design will be included. Papers reporting information regarding adherence in the context of prescribed therapeutic exercise and PA delivered via tele-rehabilitation for pwMS will be included. Information relating to adherence may comprise; methods of reporting adherence, adherence levels (e.g. exercise diaries, pedometers), investigation of pwMS 'and therapists 'experiences of adherence or a discussion of adherence. Eligibility criteria and a custom data extraction form will be piloted on a sample of papers. Quality assessment of included studies will use Critical Appraisal Skills Programme checklists. Data analysis will involve categorisation, enabling findings relating to study characteristics and research questions to be presented in narrative and tabular format.

Ethics and dissemination: Ethical approval was not required for this protocol. Findings will be submitted to a peer reviewed journal and presented at conferences. Consultation with pwMS and clinicians will help to identify other dissemination methods.

Protocol registration: www.osf.io

For peer review only - http://bmjopen.bmj.com/site/about/guidelines.xhtml

ARTICLE SUMMARY:

Strengths and limitations of this study:

- This scoping review methodology is well suited to the broad research aim, enabling an overview of the evidence relating to adherence to exercise and PA interventions delivered via tele-rehabilitation for pwMS.
- The SARS-CoV-2 pandemic has resulted in a rapid increase in research into tele-rehabilitation delivery of exercise and PA interventions over the past 24 months, therefore this review is timely in consolidating current evidence and identifying gaps within the research.
- Decisions regarding study selection, data extraction and quality appraisal will be undertaken by one reviewer, however to reduce the risk of personal bias, a second reviewer will check a percentage of these decisions.
- The inclusion of critical appraisal of studies will aid the interpretation of results and help to identify gaps within the research and the quality of the evidence base.

INTRODUCTION:

Adherence is an important predictor of health outcomes [1] with higher adherence levels associated with improved treatment success across a range of healthcare interventions and patient populations.[2-4] Adherence to treatment regimes has the potential to impact healthcare costs; one striking example is where the NHS could save £500 million annually if medication adherence was improved in five key health conditions.[5] This example illustrates the same impact non-adherence may have on other treatment intervention costs, including those relevant to this review in exercise and physical activity (PA) programmes.

Adherence is often used interchangeably with terms such as compliance, participation, and concordance,[6] despite the different meanings of these words.[7] Within this paper the term adherence will be used, as defined by The World Health Organisation (WHO); "the extent to which a person's behaviour; taking medication, following a diet, and/or executing lifestyle changes, corresponds with agreed recommendations from a health care provider".[1](p.3) For clarity within this paper, the WHO's definitions of exercise and PA will be utilised, with the term physical rehabilitation describing physical exercise and PA programmes prescribed with a therapeutic purpose. PA is defined by WHO as "any bodily movement produced by skeletal muscles that requires energy expenditure",[8](p.vii), and exercise as a subcategory of PA which is "planned, structured, repetitive, purposeful in the sense that the improvement or maintenance of one or more components of physical fitness is the objective".[8](p.vi) Throughout this paper, the term tele-rehabilitation will refer to the range of technologies which are being used as the method of communication between the rehabilitation professional and patient.[9]

Although the beneficial effects of physical rehabilitation are well documented,[10] adherence to such programmes remains problematic with adherence levels to home based unsupervised exercise reported to be as low as 30%.[11] As adherence is a key predictor of

For peer review only - http://bmjopen.bmj.com/site/about/guidelines.xhtml

the effectiveness of exercise programmes,[7] there is a clear need to improve adherence to physical rehabilitation programmes to maximise clinical outcomes. However without a gold standard way to measure adherence to unsupervised exercise [12] and a lack of validated measures,[13] it can be difficult to interpret study results;[4] a poor outcome could be due to poor adherence or a genuine lack of intervention efficacy.

To improve patient adherence to physical rehabilitation, some studies have integrated behavioural change techniques (BCTs) into their programmes.[14-16] BCTs are the active ingredient, or unit of change, within behavioural interventions used to modify a specified behaviour.[14, 17] One typical BCT for physical rehabilitation would be setting an exercise behaviour goal.[16] The integration of BCTs into physical rehabilitation programmes has elicited positive results across clinical populations including people living with Multiple Sclerosis (pwMS).[14-16, 18, 19]

Multiple Sclerosis (MS) is a progressive condition of the central nervous system, affecting over 130,000 people in the UK.[20] Typically, people experience a myriad of sensory, cognitive and motor impairments with subsequent limitations in function,[21, 22] which can impact negatively upon engagement in exercise and physical activity.[23] The benefits of exercise for improving symptoms, functional abilities, and quality of life for pwMS are well established [24] and physical rehabilitation programmes are frequently prescribed to pwMS.[20] However, pwMS are less physically active than the general population [24] and often experience difficulties in attending out-patient rehabilitation appointments,[25] limiting their access to physical rehabilitation advice and prescription.

Tele-rehabilitation has been proposed as a potential solution for patients who have difficulty in attending out-patient rehabilitation services,[26] enabling rehabilitation to be provided remotely through the use of information and communication technologies.[27] During the SARS-CoV-2 pandemic, tele-rehabilitation has been promoted within a wider rehabilitation context as a solution to not being able to provide out-patient healthcare [28] and its use is likely to continue in clinical practice in line with the Department of Health's Long Term Plan.[29]

Tele-rehabilitation which uses technology as the method of communication between the rehabilitation professional and patient [9] e.g. video conferencing is differentiated from the use of technology as the physical rehabilitation intervention (e.g. games console) within this review. This is an important distinction when attempting to evaluate adherence to physical rehabilitation programmes, as factors affecting adherence may differ with these different types of exercise provision.[12,30] This review is interested in exploring whether adherence levels and the effectiveness of BCTs delivered via tele-rehabilitation, within the context of a physical rehabilitation programme, may differ to those delivered in face-to-face settings.

Studies investigating the use of tele-rehabilitation to deliver exercise programmes and interventions to increase PA for pwMS have sought participants' [31-34] and therapists' [32] views on the programmes, however the authors are not aware of any reviews that have provided an overview of these data. Difficulties with exercise progression were reported by therapists when their communication with participants was conducted via email and online exercise diaries as they were unable to observe participants perform their exercises.[32]

For peer review only - http://bmjopen.bmj.com/site/about/guidelines.xhtml

Participants have found the tele-rehabilitation delivery acceptable,[32, 34] and that it increased flexibility [31-33] as well as reducing the transport and physical energy costs of attending appointments.[31] Systematic reviews have investigated the use of tele-rehabilitation to deliver physical rehabilitation,[35, 36] as well as the use of BCTs across tele-rehabilitation and in-person settings for pwMS.[14, 18, 19] However these reviews have either; focused on PA levels as the outcome of interest,[14, 18, 19] did not explore adherence to the intervention,[35] or included limited information detailing participants ' adherence to the prescribed physical rehabilitation programmes.[36] This scoping review aims to address this gap by specifically focusing on detailing the reported level of adherence to physical rehabilitation programmes, use of BCTs and the experiences of pwMS and therapists in adhering to these tele-rehabilitation delivered programmes.

METHODS AND ANALYSIS:

A scoping review methodology is particularly relevant in emerging fields [37] such as tele-rehabilitation; mapping the extent and nature of research and identifying research gaps.[38] The methods developed for this scoping review are based on the 6 stage framework described by Arksey and O'Malley [38] and Levac et al.[37] This protocol is reported in line with the Preferred Reporting Items for Systematic Reviews and Meta Analyses Extension for Scoping Reviews (PRISMA-ScR).[39]

Stage 1: Identifying the research question

This scoping review aims to provide an overview of the literature regarding adherence to physical rehabilitation delivered via tele-rehabilitation for pwMS. The specific research questions identified are:

- What levels of adherence are reported by studies prescribing physical rehabilitation delivered via tele-rehabilitation for pwMS?
- To what degree are valid and reliable measures of adherence used within studies? Is there evidence of integration of BCTs within the physical rehabilitation programmes prescribed, and how well is this reported?
- Is there any evidence that integrated BCTs have influenced levels of adherence to the prescribed physical rehabilitation programmes?
- What are the reported experiences of pwMS and physical rehabilitation prescribers regarding adherence to physical rehabilitation?

Stage 2: Identifying relevant studies

Scoping searches were utilised to identify free text and controlled subject heading terms for the population, intervention and outcome, and a draft search strategy for Medline (Ovid SP) was developed (strategy 1). As outcomes may not be reported within the title and abstract or picked up by the controlled vocabulary function,[40] a further search strategy (strategy 2) without terms relating to outcomes (i.e. adherence) was devised with assistance from an information specialist. The two strategies were then compared for duplicates. All papers identified in search strategy 1, were also identified in search strategy 2, however search strategy 2 identified further relevant papers that were not identified by the first strategy. A

For peer review only - http://bmjopen.bmj.com/site/about/guidelines.xhtml

search strategy without terms relating to outcomes will therefore be utilised within the scoping review to ensure that relevant papers are not missed.

The search strategies will be adapted for the following databases: Embase (OvidSP), Cinahl (EBSCOhost), The Health Management Information Consortium (HMIC) Database, ProQuest Dissertations and Theses Global (PQDT), Pedro, Cochrane central register of controlled trials, US National Library of Medicine Registry of Clinical Trials, World Health Organisation International Clinical Trials Registry Platform portal. Databases will be searched from 1998 to present day to reflect the start of the scientific publication of tele-rehabilitation studies.[41]

To identify relevant papers not included in bibliographic databases, the following organisation and associated conference websites will be searched: MS Society, MS Trust, Chartered Society of Physiotherapy, UK Society of Behavioural Medicine, International Society of Behavioural Medicine, Open Grey, National MS Society, Rehabilitation in MS (RIMS) and European Committee for Treatment and Research in MS (ECTRIMS).

A search log will be completed to record searches including the source and dates covered, platform, date of search and number of records yielded. Following study selection, the reference lists of all included studies will be searched for additional relevant papers.

Stage 3: Study selection

Papers reporting information relating to adults (18+) with MS will be included. If mixed populations are included within a study, the study will be included if separate data are reported for adult participants with MS. Information on physical rehabilitation; physical exercises, or PA prescribed for a therapeutic purpose must be included, whether this relates to individual or group delivery, as part of a multifactorial rehabilitation programme or a single intervention. Papers describing tele-rehabilitation delivery of the physical rehabilitation programme meeting Laver et al's [9] description will be included, whether the tele-rehabilitation is used as the sole programme delivery method or in combination with another method such as face-to-face.

The review will include papers reporting information relating to the concept of adherence as defined by the WHO.[1] Information relating to adherence may comprise; a method of reporting adherence, adherence levels (e.g. exercise diaries, pedometers, questionnaires, measurement scales), investigation of pwMS' experiences of adherence or a discussion of adherence.

Study protocols will be excluded, with papers of all other study designs included. In order to minimise language bias, studies in all languages will be eligible with relevant studies translated to English where possible; any that we are unable to translate will be excluded from the review and reason for exclusion noted as language.

Search results will be downloaded into EndNote and duplicates removed. If there are multiple reports of the same study, these will be compared to ensure that adequate

For peer review only - http://bmjopen.bmj.com/site/about/guidelines.xhtml

information is obtained and the study's results are only used once. The study selection process will be piloted independently by two team members on 25 studies to check the interpretation of the eligibility criteria and consistency of use. Following the piloting process, the eligibility criteria may be refined.

One reviewer will screen the titles and abstracts of papers, with 10% checked independently by a second reviewer. Papers appearing to meet the eligibility criteria and those where it is unclear from the title and abstract as to whether the criteria have been met, will have their full text screened by one reviewer, with 10% screened by a second reviewer. Discrepancies between the two reviewers at any stage of the screening process will be resolved via consensus and discussion with a third person.

The number of studies excluded at both stages of screening alongside reasons for exclusion will be recorded to enable completion of a PRISMA flow diagram and narrative summary of the screening decision process.

Stage 4: Charting the data

Data extraction will be piloted on five included studies independently by two reviewers, with processes amended as required. This piloting process will ensure that the instructions are applied consistently and the planned data extraction allows relevant characteristics of each study to be presented in the review alongside a quality assessment and details relating to the research questions and review aim.

Following the methodology in other scoping reviews and protocols,[42,43] data will be extracted into a custom data extraction form by one reviewer. We will then differ from other methodologies [42,43] as 10% of our data extraction will be checked by a second reviewer. Disagreements will be resolved by consensus and discussion with a third reviewer. The date of extraction and reviewer undertaking the extraction will be recorded alongside key information regarding the paper including: Author; year and country of publication; aim and objectives of paper; participant / population information; Methods (including study design, blinding, randomisation, time points of data collection); Intervention description (including details of tele-rehabilitation methods, exercise or PA interventions, BCTs, comparators); Outcomes and results (including adherence measurement tools e.g. session attendance or exercise diaries, information relating to participants 'experiences); key findings and discussion points relating to the research questions. Where relevant information is missing from articles, authors will be contacted for further information where possible.

Stage 5: Collating, summarising and reporting the results

This stage will involve three distinct steps; analysis of the data, reporting of the results and outcome in relation to the research questions; and consideration of the meaning of the findings.[37] The methodological quality of included studies will be assessed independently by one reviewer with 10% of decisions checked by a second reviewer, both using tools from

For peer review only - http://bmjopen.bmj.com/site/about/guidelines.xhtml

the Critical Appraisal Skills Programme suite.[44] A third reviewer will be available to mediate any disagreements.

Analysis of the data:

The planned analysis of data will occur in two stages. Firstly an analysis of the data extracted from all included studies with regard to their characteristics and quality appraisal will be undertaken. Secondly the research questions will be used to structure and organise an analysis of study findings, outcomes and interventions. Where appropriate, frequency counts will be utilised for information including study design, use of adherence measures (e.g. exercise diary), reporting of adherence levels and intervention characteristics (e.g. type of tele-rehabilitation and BCTs utilised). Data relating to participant adherence levels will be analysed through frequency counts to identify the number of studies reporting a level of adherence and using each measurement tool. Data relating to participants' and prescribers' experiences of adherence within studies will be analysed through the use of categories and themes.

Reporting of the results and outcome in relation to the research questions:

The characteristics of all included studies incorporating study design, population, interventions and quality appraisal will be presented in a table and narrative summary. Extracted data and the review's findings will be presented in a narrative summary addressing each of the research questions, with the use of tables and diagrams to aid interpretation where appropriate. Data relating to the level of adherence described by studies (research question 1) will be reported with reference to the measurement tool used (research question 2) to provide greater context and appropriate grouping of data relating to adherence levels.

Consideration of the meaning of the findings:

Findings will be summarised in the context of whether the review's aims have been met and research questions answered. Discussion of the key findings will be included alongside identification of gaps in current knowledge and corresponding implications for future research. The quality assessment of papers may aid the interpretation of results [45] and identification of gaps within the literature.[37]

Stage 6: Consultation

It is planned that consultation will involve pwMS and therapists working with pwMS in community settings (those working with pwMS in their own homes and out-patient settings). The aims of the planned consultation are to share and discuss preliminary findings, inform future research and develop dissemination methods. The discussion of preliminary findings provides the opportunity to discuss findings within the context of pwMS and clinicians' experiences of adherence to physical rehabilitation delivered via tele-rehabilitation.

Patient and Public Involvement and Engagement:

Patient and Public Involvement and Engagement (PPIE) was sought in the development of the review's aims and research questions alongside the identification of possible

For peer review only - http://bmjopen.bmj.com/site/about/guidelines.xhtml

dissemination methods to utilise following the completion of the review. The PPIE was undertaken with a group of four pwMS who provided written feedback on a lay summary of the scoping review protocol. Videoconferencing was then used with three pwMS from this group to explore further their priorities and experience of the use of tele-rehabilitation. This included discussions regarding their general experiences of tele-rehabilitation use alongside specific questions regarding adherence when therapeutic physical rehabilitation programmes have been delivered via tele-rehabilitation.

The feedback regarding the importance of the proposed area of research was useful in shaping the context of the review and its research questions. The PPIE feedback has been incorporated into the protocol through formulation of a research question relating to the experiences of pwMS and identification of dissemination methods as detailed below.

For peer review only

ETHICS AND DISSEMINATION:

Ethical approval was not required in the development of this protocol as the planned methodology involves the review of publicly available data.

The findings of the review will be submitted to a peer reviewed journal and for presentation at conferences. Further dissemination to clinicians working with pwMS will be guided by the consultation process. PPIE in the design of this protocol identified discussion with MS charities e.g. MS Trust regarding use of their social media or website platforms and newsletters to promote key findings as an important potential method of dissemination to pwMS, their families and carers. Further PPIE input through the consultation stage of the review may identify further appropriate dissemination methods for pwMS.

AUTHOR CONTRIBUTIONS:

G. Goldsmith, J. Bollen , J. Freeman, V. Salmon , S. Dean
Idea for scoping review (GG) with help from all authors in it's development. Refinement of aims and objectives by GG, JB, JF and SD. Search strategy written by GG with input from PenARC Information Specialist and all authors. Methods drafted by GG with input from all authors and PenARC search and review clinic team. All authors read and commented on the final draft.
PPIE contributors: Thank you to Lynn Tatnell, Jessica Hole and Melanie Guy and other PPIE contributors for their time and help in reviewing the protocol and their contribution to it's development. Thank you to PenARC PPIE team for their help and support in arranging the PPIE contributions to this protocol.

FUNDING STATEMENT:

This work was supported by a Health Education England and the National Institute of Health (NIHR) Research South West Internship funding award (GG). SD's time is partially supported by the NIHR Applied Research Collaboration South West Peninsula. The views expressed in

this publication are those of the author(s) and not necessarily those of the NIHR or the Department of Health and Social Care.

COMPETING INTEREST STATEMENT:

Geraldine Goldsmith received funding through a HEE/NIHR ICA Internship (Nov 2020 – April 2021).

DATA SHARING STATEMENT:

Data sharing not applicable as no datasets generated and/or analysed for this study.

FULL REFERENCES:

1. World Health Organisation Eduardo Sabaté (ed.) *Adherence to long term therapies: evidence for action* World Health Organisation (2003) Available from: https://apps.who.int/iris/bitstream/handle/10665/42682/9241545992.pdf?sequence=1&isAllowed=y (accessed 13 Dec 2021)

2 Neter E, Wolkowitz A, Glass-Marmor L, et al. Multiple modality approach to assess adherence to medications across time in Multiple Sclerosis. *Mult Scler Relat Disord*. 2020;May:40:101951. doi: 10.1016/j.msard.2020.101951. [published online 15 Jan 2020]

3 Quintana-Navarro GM, Alcala-Diaz JF, Lopez-Moreno J, et al. Long-term dietary adherence and changes in dietary intake in coronary patients after intervention with a Mediterranean diet or a low-fat diet: the CORDIOPREV randomized trial. *Eur J Nutr*. 2020;59(5):2099-2110. doi: 10.1007/s00394-019-02059-5. [published online 24 Jul 2019]

4 Holt CJ, McKay CD, Truong LK, et al. Sticking to It: A Scoping Review of Adherence to Exercise Therapy Interventions in Children and Adolescents With Musculoskeletal Conditions. *J Orthop Sports Phys Ther.* 2020;50(9):503-515. doi: 10.2519/jospt.2020.9715. [published online 1 Aug 2020]

5. Trueman, P; Taylor, DG; Lowson K, et al. *Evaluation of the Scale, Cause and Costs of Waste Medicines. Final Report*. York Health Economics Consortium, The School of Pharmacy, University of London (2010). Available from: https://discovery.ucl.ac.uk/id/eprint/1350234/1/Evaluation_of_NHS_Medicines_Waste__web_publication_version.pdf (accessed 13 Dec 2021)

6. Dennett R, Madsen LT, Connolly L, et al. Adherence and drop-out in randomized controlled trials of exercise interventions in people with multiple sclerosis: A systematic review and meta-analyses. *Mult Scler Relat Disord*. 2020;43:102169. doi: 10.1016/j.msard.2020.102169. [published online 19 May 2020]

For peer review only - http://bmjopen.bmj.com/site/about/guidelines.xhtml

7. Bailey DL, Holden MA, Foster NE, et al Defining adherence to therapeutic exercise for musculoskeletal pain: a systematic review *Br J Sports Med* 2020;54:326-331. doi: 10.1136/bjsports-2017-098742. [published online 6 June 2018]

8. World Health Organisation *WHO Guidelines on Physical Activity and Sedentary Behaviour* The World Health Organisation 2020 Available from: https://apps.who.int/iris/bitstream/handle/10665/336656/9789240015128-eng.pdf?sequence=1&isAllowed=y (accessed 21 Nov 2021)

9. Laver KE, Adey-Wakeling Z, Crotty M, et al. Telerehabilitation services for stroke. *Cochrane Database of Syst Rev*. 2020(1). doi: 10.1002/14651858.CD010255.pub3.

10. Department of Health and Social Care *UK Chief Medical Officers' Physical Activity Report* 2019 https://assets.publishing.service.gov.uk/government/uploads/system/uploads/attachment_data/file/832868/uk-chief-medical-officers-physical-activity-guidelines.pdf (accessed 21 Nov 2021)

11. Essery R, Geraghty AW, Kirby S, et al. Predictors of adherence to home-based physical therapies: a systematic review. *Disabil Rehabil*. 2017;39(6):519-534. doi: 10.3109/09638288.2016.1153160. [published online 21 April 2016]

12. Argent, R, Daly A, Caulfield B. Patient Involvement With Home-Based Exercise Programs: Can Connected Health Interventions Influence Adherence? *JMIR Mhealth Uhealth* 2018;6(3):e47. doi: **10.2196/mhealth.8518** [published online 1 Mar 2018]

13. Bollen JC, Dean SG, Siegert RJ, et al. A systematic review of measures of self-reported adherence to unsupervised home-based rehabilitation exercise programmes, and their psychometric properties. *BMJ open* 2014;4(6):e005044. doi: 10.1136/bmjopen-2014-005044

14. Casey B, Coote S, Hayes S, et al. Changing Physical Activity Behavior in People With Multiple Sclerosis: A Systematic Review and Meta-Analysis. *Arch Phys Med Rehabil*. 2018;99(10):2059-2075. doi: 10.1016/j.apmr.2017.12.013. [published online 11 Jan 2018]

15. Meade LB, Bearne LM, Sweeney LH, et al. Behaviour change techniques associated with adherence to prescribed exercise in patients with persistent musculoskeletal pain: Systematic review. *Br J Health Psychol*. 2019;24(1):10-30. doi: 10.1111/bjhp.12324. [published online 17 June 2018]

16. Willett M, Duda J, Fenton S, et al. Effectiveness of behaviour change techniques in physiotherapy interventions to promote physical activity adherence in lower limb osteoarthritis patients: A systematic review. *PLoS One*. 2019;14(7):e0219482. doi: 10.1371/journal.pone.0219482.

17. Michie, S., van Stralen, M.M., West, R. (2011) The behaviour change wheel: A new method for characterising and designing behaviour change interventions *Implement Sci.* 2011;6:42. doi: 10.1186/1748-5908-6-42

For peer review only - http://bmjopen.bmj.com/site/about/guidelines.xhtml

18. Coulter EH, Bond S, Dalgas U, et al. The effectiveness of interventions targeting physical activity and/or sedentary behaviour in people with Multiple Sclerosis: a systematic review. *Disabil Rehabil*. 2020;42(5):594-612. doi: 10.1080/09638288.2018.1503737. [published online 5 Oct 2018]

19. Sangelaji B, Smith CM, Paul L, et al. The effectiveness of behaviour change interventions to increase physical activity participation in people with multiple sclerosis: a systematic review and meta-analysis. *Clin Rehabil*. 2016;30(6):559-76. doi: 10.1177/0269215515595274. [published online 21 July 2015]

20. MS Society *What is MS?* Available from: https://www.mssociety.org.uk/about-ms/what-is-ms [accessed: 28 Dec 2020]

21. Rietberg MB, Brooks D, Uitdehaag BM, et al. Exercise therapy for multiple sclerosis. *Cochrane Database Syst Rev*.2005(1):CD003980. doi:10.1002/14651858.CD003980.pub2.

22. Amatya B, Khan F, Galea M. Rehabilitation for people with multiple sclerosis: an overview of Cochrane Reviews. *Cochrane Database of Syst Rev*. 2019(1). doi: 10.1002/14651858.CD12732.pub2

23. Stuifbergen AK, Blozis SA, Harrison TC, et al. Exercise, functional limitations, and quality of life: a longitudinal study of persons with multiple sclerosis. *Arch Phys Med Rehabil* 2006;87:935–943. doi: 10.1016/j.apmr.2006.04.003.

24. Motl RW, Sandroff BM, Kwakkel G, et al. Exercise in patients with multiple sclerosis. *Lancet neurol*. 2017;16(10):848-56. doi: 10.1016/S1474-4422(17)30281-8. [12 Sep 2017]

25. Shaw MT, Best P, Frontario A, et al. Telerehabilitation benefits patients with multiple sclerosis in an urban setting. *J Telemed Telecare*. 2021;27(1):39-45. doi: 10.1177/1357633X19861830. [published online 15 Jul 2019]

26. Rimmer JH, Thirumalai M, Young HJ, et al. Rationale and design of the tele-exercise and multiple sclerosis (TEAMS) study: A comparative effectiveness trial between a clinic- and home-based telerehabilitation intervention for adults with multiple sclerosis (MS) living in the deep south. *Contemp Clin Trials*. 2018;71:186-193. doi: 10.1016/j.cct.2018.05.016. [published online 30 May 2018]

27. Brennan DM, Mawson S, Brownsell S. Telerehabilitation: enabling the remote delivery of healthcare, rehabilitation, and self management. *Stud Health Technol Inform*. 2009;145:231-48. Available from: https://pubmed.ncbi.nlm.nih.gov/19592797/

28. Signal N, Martin T, Leys A,et al. Implementation of telerehabilitation in response to COVID-19: lessons learnt from neurorehabilitation clinical practice and education. *NZ J Physiother*2020;48:117-26. Available from: https://doi.org/10.15619/NZJP/48.3.03

29. Department of Health *NHS Long Term Plan* 2010 Available from: https://www.longtermplan.nhs.uk/online-version/chapter-5-digitally-enabled-care-will-go-mainstream-across-the-nhs/ (accessed 1 June 2020)

30. Valenzuela T, Okubo Y, Woodbury A, et al. Adherence to Technology-Based Exercise Programs in Older Adults: A Systematic Review. *J Geriatr Phys Ther*. 2018;41(1):49-61. doi: 10.1519/JPT.0000000000000095

31. Paul L, Coulter EH, Miller L, et al. Web-based physiotherapy for people moderately affected with Multiple Sclerosis; quantitative and qualitative data from a randomized, controlled pilot study. *Clin Rehabil*. 2014;28(9):924-35. doi: 10.1177/0269215514527995. [published online 1 Apr 2014]

32. Paul L, Renfrew L, Freeman J, et al. Web-based physiotherapy for people affected by multiple sclerosis: a single blind, randomized controlled feasibility study. *Clin Rehabil*. 2019Mar;33(3):473-84. doi: 10.1177/0269215518817080. [published online 4 Dec 2018]

33. Dennett R, Coulter E, Paul L, et al. A qualitative exploration of the participants ' experience of a web-based physiotherapy program for people with multiple sclerosis: Does it impact on the ability to increase and sustain engagement in physical activity?. *Disabil Rehabil*. 2020;8;42(21):3007-14. doi: 10.1080/09638288.2019.1582717. [published online 23 Mar 2019]

34. Dlugonski D, Motl RW, McAuley E. Increasing physical activity in multiple sclerosis: replicating Internet intervention effects using objective and self-report outcomes. *J Rehabil Res Dev*. 2011;48(9):1129-36. doi: 10.1682/jrrd.2010.09.0192.

35. Rintala A, Hakala S, Paltamaa J, et al. Effectiveness of technology-based distance physical rehabilitation interventions on physical activity and walking in multiple sclerosis: a systematic review and meta-analysis of randomized controlled trials. *Disabil Rehabil*. 2018;40(4):373-387. doi: 10.1080/09638288.2016.1260649. [published online 15 Dec 2016]

36. Dennett, R., Gunn, H., Freeman, J.A. Effectiveness of and User Experience With Web-Based Interventions in Increasing Physical Activity Levels in People With Multiple Sclerosis: A Systematic Review, *Phys Ther*, 2018;98(8)679-690 https://doi.org/10.1093/ptj/pzy060

37. Levac D, Colquhoun H, & O'Brien K.K. Scoping studies: advancing the methodology. *Implementation Sci* 2010;5,69. https://doi.org/10.1186/1748-5908-5-69

38. Arksey, H. & O'Malley, L. Scoping studies: towards a methodological framework *Int J Soc Res Methodol* 2005;8(1):19-32, doi: 10.1080/1364557032000119616

39. Tricco, AC, Lillie, E, Zarin, W, et al. PRISMA extension for scoping reviews (PRISMA-ScR): checklist and explanation. *Ann Intern Med*. 2018,169(7):467-473. doi: 10.7326/M18-0850. [published online 4 Sep 2018]

For peer review only - http://bmjopen.bmj.com/site/about/guidelines.xhtml

40. Lefebvre C, Glanville J, Briscoe S, et al. Chapter 4: Searching for and selecting studies. In: Higgins JPT, Thomas J, Chandler J, Cumpston M, Li T, Page MJ, Welch VA (eds). *Cochrane Handbook for Systematic Reviews of Interventions* version 6.1 (updated September 2020). Cochrane, 2020 Available from www.training.cochrane.org/handbook. (accessed 13 Dec 2021)

41. Peretti A, Amenta F, Tayebati SK, et al. Telerehabilitation: Review of the State-of-the-Art and Areas of Application. *JMIR Rehabil Assist Technol*. 2017;4(2):e7. doi: 10.2196/rehab.7511.

42. Parker M, Fang X, Self-Brown SR, et al. Establishing how social capital is studied in relation to cardiovascular disease and identifying gaps for future research-A scoping review protocol. *PLoS one*. 2021;8;16(4):e0249751. doi: 10.1371/journal.pone.0249751.

43. Herman C, Mayer K, Sarwal A. Scoping review of prevalence of neurologic comorbidities in patients hospitalized for COVID-19. *Neurology*. 2020;14;95(2):77-84. doi: 10.1212/WNL.0000000000009673. [published online 28 Apr 2020]

44. Critical Appraisal Skills Programme *Checklists* Available from: https://casp-uk.net/casp-tools-checklists/ (accessed 27.12.22)

45. Centre for Reviews and Dissemination *Systematic reviews. CRD's guidance for undertaking reviews in healthcare CRD* University of York. 2008 Available from https://www.york.ac.uk/media/crd/Systematic_Reviews.pdf (accessed 14.12.2020)

WORD COUNT: 3031

only

For peer review only - http://bmjopen.bmj.com/site/about/guidelines.xhtml

BMJ Open

Adherence to physical rehabilitation delivered via tele-rehabilitation for people with Multiple Sclerosis: a scoping review protocol

Journal:	*BMJ Open*
Manuscript ID	bmjopen-2022-062548.R1
Article Type:	Protocol
Date Submitted by the Author:	23-Jan-2023
Complete List of Authors:	Goldsmith, Geraldine; University of Exeter<br>Bollen, Jessica; PenCLAHRC University of Exeter Medical School,<br>Salmon , Victoria ; University of Exeter,<br>Freeman, Jennifer; Peninsula Allied Hlth Ctr, Faculty of Health and Human Sciences<br>Dean, Sarah; PenCLAHRC University of Exeter Medical School,
**Primary Subject Heading**:	Rehabilitation medicine
Secondary Subject Heading:	Neurology
Keywords:	Multiple sclerosis < NEUROLOGY, Telemedicine < BIOTECHNOLOGY & BIOINFORMATICS, REHABILITATION MEDICINE

SCHOLARONE™
Manuscripts

Adherence to physical rehabilitation delivered via tele-rehabilitation for people with Multiple Sclerosis: a scoping review protocol

Corresponding author:
Geraldine Goldsmith
Exeter Community Neuro-rehabilitation Team, Poltimore Centre, Exeter Community Hospital, Hospital Lane, Exeter, EX1 3RB
g.goldsmith@exeter.ac.uk

Co-authors:

Dr Jessica Bollen
University of Exeter Medical School
Exeter, UK

Dr Victoria Salmon
University of Exeter Medical School
Exeter, UK

Professor Jennifer Freeman
Faculty of Health, School of Health Professions
University of Plymouth
Plymouth, UK

Professor Sarah Dean
University of Exeter Medical School
Exeter, UK

Word count: 3286

For peer review only - http://bmjopen.bmj.com/site/about/guidelines.xhtml

ABSTRACT:

Introduction: Using tele-rehabilitation methods to deliver exercise, physical activity (PA) and behaviour change interventions for people with Multiple Sclerosis (pwMS) has increased in recent years, especially since the SARS-CoV-2 pandemic. This scoping review aims to provide an overview of the literature regarding adherence to therapeutic exercise and PA delivered via tele-rehabilitation for pwMS.

Methods and analysis: Frameworks described by Arksey and O'Malley (2005) and Levac *et al* (2010) underpin the methods. The following databases will be searched from 1998 to present: Medline (Ovid), Embase (Ovid), Cinahl (EBSCOhost), Health Management Information Consortium Database, ProQuest Dissertations and Theses Global, Pedro, Cochrane central register of controlled trials, US National Library of Medicine Registry of Clinical Trials, World Health Organisation International Clinical Trials Registry Platform portal, and The Cochrane Database of Systematic Reviews. To identify papers not included in databases, relevant websites will be searched. Searches are planned for 2023. With the exception of study protocols, papers of any study design will be included. Papers reporting information regarding adherence in the context of prescribed therapeutic exercise and PA delivered via tele-rehabilitation for pwMS will be included. Information relating to adherence may comprise; methods of reporting adherence, adherence levels (e.g. exercise diaries, pedometers), investigation of pwMS 'and therapists 'experiences of adherence or a discussion of adherence. Eligibility criteria and a custom data extraction form will be piloted on a sample of papers. Quality assessment of included studies will use Critical Appraisal Skills Programme checklists. Data analysis will involve categorisation, enabling findings relating to study characteristics and research questions to be presented in narrative and tabular format.

Ethics and dissemination: Ethical approval was not required for this protocol. Findings will be submitted to a peer reviewed journal and presented at conferences. Consultation with pwMS and clinicians will help to identify other dissemination methods.

Protocol registration: www.osf.io

For peer review only - http://bmjopen.bmj.com/site/about/guidelines.xhtml

ARTICLE SUMMARY:

Strengths and limitations of this study:

- The SARS-CoV-2 pandemic has resulted in a rapid increase in research into tele-rehabilitation delivery of exercise and PA interventions, with this scoping review methodology well suited to the broad and emerging nature of this evidence.
- Decisions regarding study selection, data extraction and quality appraisal will be undertaken by one reviewer, however to reduce the risk of bias, a second reviewer will check 20% of these decisions.
- The inclusion of critical appraisal of studies will aid the interpretation of results and help to identify gaps within the research and the quality of the evidence base.
- The search strategy includes peer-reviewed evidence from electronic databases alongside organisation websites and conference proceedings.
- Patient and Public Involvement and Engagement was sought in the development of this scoping review, with a plan for further consultation with stakeholders during the review process.

INTRODUCTION:

Adherence is an important predictor of health outcomes [1] with higher adherence levels associated with improved treatment success across a range of healthcare interventions and patient populations.[2-4] Adherence to treatment regimes has the potential to impact healthcare costs; one striking example is where the NHS could save £500 million annually if medication adherence was improved in five key health conditions.[5] This example illustrates the same impact non-adherence may have on other treatment intervention costs, including those relevant to this review in exercise and physical activity (PA) programmes.

Adherence is often used interchangeably with terms such as compliance, participation, and concordance,[6] despite the different meanings of these words.[7] The issue is complicated further within therapeutic exercise prescription where various parameters of adherence i.e. the aspects of adherence that can be measured, have been identified.[7] These parameters include the frequency of exercise (e.g. repetitions or sets), the quality of the exercises performed, but also attendance which is only a proxy marker of actually doing the exercises. These authors suggest that whilst several parameters may be relevant to therapeutic exercise, there is a lack of consensus regarding the relevance and importance in specific contexts.[7]

For clarity and to encompass the multifaceted nature of the concept and parameters of adherence, within this paper the term adherence will be used, as defined by The World Health Organisation (WHO); “the extent to which a person's behaviour; taking medication, following a diet, and/or executing lifestyle changes, corresponds with agreed recommendations from a health care provider”.[1](p.3) For clarity within this paper, the WHO’s definitions of exercise and PA will be utilised, with the term physical rehabilitation describing physical exercise and PA programmes prescribed with a therapeutic purpose. PA is defined by WHO as “any bodily movement produced by skeletal muscles that requires energy expenditure”,[8](p.vii), and exercise as a subcategory of PA which is “planned,

For peer review only - http://bmjopen.bmj.com/site/about/guidelines.xhtml

structured, repetitive, purposeful in the sense that the improvement or maintenance of one or more components of physical fitness is the objective".[8](p.vi) Throughout this paper, the term tele-rehabilitation will refer to the range of technologies which are being used as the method of communication between the rehabilitation professional and patient,[9] allowing for a double communication loop, whereby the patient's performance is monitored and relayed to the clinician who can then respond with appropriate feedback.[10]

Although the beneficial effects of physical rehabilitation are well documented,[11] adherence to such programmes remains problematic with adherence levels to home based unsupervised exercise reported to be as low as 30%.[12] As adherence is a key predictor of the effectiveness of exercise programmes,[7] there is a clear need to improve adherence to physical rehabilitation programmes to maximise clinical outcomes. However without a gold standard way to measure adherence to unsupervised exercise [13] and a lack of validated measures,[14] it can be difficult to interpret study results;[4] a poor outcome could be due to poor adherence or a genuine lack of intervention efficacy.

To improve patient adherence to physical rehabilitation, some studies have integrated behavioural change techniques (BCTs) into their programmes.[15-17] BCTs are the active ingredient, or unit of change, within behavioural interventions used to modify a specified behaviour.[15, 18] One typical BCT for physical rehabilitation would be setting an exercise behaviour goal.[17] The integration of BCTs into physical rehabilitation programmes has elicited positive results across clinical populations including people living with Multiple Sclerosis (pwMS).[15-17, 19, 20]

Multiple Sclerosis (MS) is a progressive condition of the central nervous system, affecting over 130,000 people in the UK.[21] Typically, people experience a myriad of sensory, cognitive and motor impairments with subsequent limitations in function,[22, 23] which can impact negatively upon engagement in exercise and physical activity.[24] The benefits of exercise for improving symptoms, functional abilities, and quality of life for pwMS are well established [25] and physical rehabilitation programmes are frequently prescribed to pwMS.[21] However, pwMS are less physically active than the general population [25] and often experience difficulties in attending out-patient rehabilitation appointments,[26] limiting their access to physical rehabilitation advice and prescription.

Tele-rehabilitation has been proposed as a potential solution for patients who have difficulty in attending out-patient rehabilitation services,[27] and has been promoted within a wider rehabilitation context during the SARS-CoV-2 pandemic [28] with its use likely to continue in clinical practice in line with the Department of Health's Long Term Plan.[29] Within the context of rehabilitation, the 'double communication loop' is an essential element of the telerehabilitation as it allows clinicians to adjust or progress a patient's exercise programme according to their performance.[10] When delivering interventions via tele-rehabilitation, the interaction between clinician and patient may be synchronous (in real time), asynchronous (not in real time) or mixed, with the feedback received by the clinician delivered online (within the intervention) or offline (with a delay).[10, 30]

Within rehabilitation, technology can be utilised in various contexts including; to enable the communication between the patient and clinician e.g. video conferencing, or as the

For peer review only - http://bmjopen.bmj.com/site/about/guidelines.xhtml

rehabilitation intervention itself e.g. using wii fit games to improve upper limb motor function. Distinguishing between these two uses of technology is important when attempting to evaluate adherence to physical rehabilitation programmes, as using technology as the rehabilitation intervention may affect adherence.[13,31] This review is interested in exploring whether adherence levels and the effectiveness of BCTs delivered via tele-rehabilitation, within the context of a physical rehabilitation programme, may differ to those delivered in face-to-face settings.

Studies investigating the use of tele-rehabilitation to deliver exercise programmes and interventions to increase PA for pwMS have sought participants' [32-35] and therapists' [33 views on the programmes, however the authors are not aware of any reviews that have provided an overview of these data. Difficulties with exercise progression were reported by therapists when their communication with participants was conducted via email and online exercise diaries as they were unable to observe participants perform their exercises.[33] Participants have found the tele-rehabilitation delivery acceptable,[33, 35] and that it increased flexibility [32-34] as well as reducing the transport and physical energy costs of attending appointments.[32] Systematic reviews have investigated the use of tele-rehabilitation to deliver physical rehabilitation,[36,37] as well as the use of BCTs across tele-rehabilitation and in-person settings for pwMS.[15,19,20] However these reviews have either; focused on PA levels as the outcome of interest,[15,19,20] did not explore adherence to the intervention,[36] or included limited information detailing participants 'adherence to the prescribed physical rehabilitation programmes.[37] This scoping review aims to address this gap by specifically focusing on detailing the reported level of adherence to physical rehabilitation programmes, use of BCTs and the experiences of pwMS and therapists in adhering to these tele-rehabilitation delivered programmes.

METHODS AND ANALYSIS:

A scoping review methodology is particularly relevant in emerging fields [38] such as tele-rehabilitation; mapping the extent and nature of research and identifying research gaps.[39] The methods developed for this scoping review are based on the 6 stage framework described by Arksey and O'Malley [39] and Levac et al.[38] This protocol is reported in line with the Preferred Reporting Items for Systematic Reviews and Meta Analyses Extension for Scoping Reviews (PRISMA-ScR).[40]

Stage 1: Identifying the research question

This scoping review aims to provide an overview of the literature regarding adherence to physical rehabilitation delivered via tele-rehabilitation for pwMS. The specific research questions identified are:

- What levels of adherence are reported by studies prescribing physical rehabilitation delivered via tele-rehabilitation for pwMS?
- To what degree are valid and reliable measures of adherence used within studies?
- Is there evidence of integration of BCTs within the physical rehabilitation programmes prescribed, and how well is this reported?

For peer review only - http://bmjopen.bmj.com/site/about/guidelines.xhtml

- Is there any evidence that integrated BCTs have influenced levels of adherence to the prescribed physical rehabilitation programmes?
- What are the reported experiences of pwMS and physical rehabilitation prescribers regarding adherence to physical rehabilitation?

Stage 2: Identifying relevant studies

Scoping searches were utilised to identify free text and controlled subject heading terms for the population, intervention and outcome, and a draft search strategy for Medline (Ovid) was developed (strategy 1). As outcomes may not be reported within the title and abstract or picked up by the controlled vocabulary function,[41] a further search strategy (strategy 2) without terms relating to outcomes (i.e. adherence) was devised with assistance from an information specialist who provided advice regarding free text terms, appropriate databases and supplementary searching. The two strategies were then compared for duplicates. All papers identified in search strategy 1, were also identified in search strategy 2, however search strategy 2 identified further relevant papers that were not identified by the first strategy. A search strategy without terms relating to outcomes will therefore be utilised within the scoping review to ensure that relevant papers are not missed. Search strategy 2 is presented within the supplementary file.

The search strategies will be adapted for the following databases: Embase (Ovid), Cinahl (EBSCOhost), Health Management Information Consortium (HMIC) Database, ProQuest Dissertations and Theses Global (PQDT), Pedro, Cochrane central register of controlled trials, US National Library of Medicine Registry of Clinical Trials, World Health Organisation International Clinical Trials Registry Platform portal. Databases will be searched from 1998 to present day to reflect the start of the scientific publication of tele-rehabilitation studies.[42]

To identify relevant papers not included in bibliographic databases, the following organisation and associated conference websites will be searched: MS Society, MS Trust, Chartered Society of Physiotherapy, UK Society of Behavioural Medicine, International Society of Behavioural Medicine, Open Grey, National MS Society, Rehabilitation in MS (RIMS) and European Committee for Treatment and Research in MS (ECTRIMS).

A search log will be completed to record searches including the source and dates covered, platform, date of search and number of records yielded. Following study selection, the reference lists of all included studies will be searched for additional relevant papers.

Stage 3: Study selection

Papers reporting information relating to adults (18+) with MS will be included. If mixed populations are included within a study, the study will be included if separate data are reported for adult participants with MS. Information on physical rehabilitation; physical exercises, or PA prescribed for a therapeutic purpose must be included, whether this relates to individual or group delivery, as part of a multifactorial rehabilitation programme or a single intervention. Papers describing tele-rehabilitation provision of the physical rehabilitation programme meeting Laver et al's [9] and Di Tella et al's [10] descriptions will be included,

For peer review only - http://bmjopen.bmj.com/site/about/guidelines.xhtml

whether the tele-rehabilitation is used as the sole programme delivery method or in combination with another method such as face-to-face.

The review will include papers reporting information relating to the concept of adherence as defined by the WHO.[1] Information relating to adherence may comprise; a method of reporting adherence, adherence levels (e.g. exercise diaries, pedometers, questionnaires, measurement scales), investigation of pwMS' or therapists' experiences of adherence or a discussion of adherence.

Study protocols will be excluded, with papers of all other study designs included. In order to minimise language bias, studies in all languages will be eligible with relevant studies translated to English where possible; any that we are unable to translate will be excluded from the review and reason for exclusion noted as language.

Search results will be downloaded into EndNote and duplicates removed. If there are multiple reports of the same study, these will be compared to ensure that adequate information is obtained and the study's results are only used once. The study selection process will be piloted independently by two team members on 25 studies to check the interpretation of the eligibility criteria and consistency of use. Following the piloting process, the eligibility criteria may be refined.

One reviewer will screen the titles and abstracts of papers, with 20% checked independently by a second reviewer. Papers appearing to meet the eligibility criteria and those where it is unclear from the title and abstract as to whether the criteria have been met, will have their full text screened by one reviewer, with 20% screened by a second reviewer. Discrepancies between the two reviewers at any stage of the screening process will be resolved via consensus and discussion with a third person. These processes meet the requirements set out by Pluddemann *et al* [43] for the study selection stage of restricted systematic reviews and reflect the method used by other authors.[44]

The number of studies excluded at both stages of screening alongside reasons for exclusion will be recorded to enable completion of a PRISMA flow diagram and narrative summary of the screening decision process.

Stage 4: Charting the data

Data extraction will be piloted on five included studies independently by two reviewers, with processes amended as required. This piloting process will ensure that the instructions are applied consistently and the planned data extraction allows relevant characteristics of each study to be presented in the review alongside a quality assessment and details relating to the research questions and review aim.

Following the methodology in other scoping reviews and protocols,[45,46] and meeting the minimum requirements for restricted systematic reviews as set out by Pluddemann et al,[43] data will be extracted into a custom data extraction form by one reviewer. To reduce data bias error, partial verification (20%) of data extraction will be undertaken by a second reviewer.[43] Disagreements will be resolved by consensus and discussion with a third

For peer review only - http://bmjopen.bmj.com/site/about/guidelines.xhtml

reviewer. The date of extraction and reviewer undertaking the extraction will be recorded alongside key information regarding the paper including: Author; year and country of publication; aim and objectives of paper; participant / population information; Methods (including study design, blinding, randomisation, time points of data collection); Intervention description (including details of tele-rehabilitation methods; synchronous/asynchronous delivery, online/offline feedback, type of technology, exercise or PA interventions, BCTs, comparators); Outcomes and results (including adherence measurement tools e.g. session attendance or exercise diaries, information relating to participants ʾexperiences); key findings and discussion points relating to the research questions. Where relevant information is missing from articles, authors will be contacted for further information where possible.

Stage 5: Collating, summarising and reporting the results

This stage will involve three distinct steps; analysis of the data, reporting of the results and outcome in relation to the research questions; and consideration of the meaning of the findings.[38] The methodological quality of included studies will be assessed independently by one reviewer with 20% of decisions checked by a second reviewer, both using tools from the Critical Appraisal Skills Programme suite.[47] A third reviewer will be available to mediate any disagreements.

Analysis of the data:
The planned analysis of data will occur in two stages. Firstly an analysis of the data extracted from all included studies with regard to their characteristics and quality appraisal will be undertaken. Secondly the research questions will be used to structure and organise an analysis of study findings, outcomes and interventions. Where appropriate, frequency counts will be utilised for information including study design, use of adherence measures (e.g. exercise diary), reporting of adherence levels and intervention characteristics (e.g. type of tele-rehabilitation and BCTs utilised). Data relating to participant adherence levels will be analysed through frequency counts to identify the number of studies reporting a level of adherence and using each measurement tool. Data relating to participants' and prescribers' experiences of adherence within studies will be analysed through the use of categories and themes.

Reporting of the results and outcome in relation to the research questions:
The characteristics of all included studies incorporating study design, population, interventions and quality appraisal will be presented in a table and narrative summary. Extracted data and the review's findings will be presented in a narrative summary addressing each of the research questions, with the use of tables and diagrams to aid interpretation where appropriate. Data relating to the level of adherence described by studies (research question 1) will be reported with reference to the measurement tool used (research question 2) and adherence parameter measured to provide greater context and appropriate grouping of data relating to adherence levels.

Consideration of the meaning of the findings:
Findings will be summarised in the context of whether the review's aims have been met and research questions answered. Discussion of the key findings will be included alongside identification of gaps in current knowledge and corresponding implications for future

For peer review only - http://bmjopen.bmj.com/site/about/guidelines.xhtml

research. The quality assessment of papers may aid the interpretation of results [48] and identification of gaps within the literature.[38]

Stage 6: Consultation

It is planned that consultation will involve pwMS and therapists working with pwMS in community settings (those working with pwMS in their own homes and out-patient settings). The aims of the planned consultation are to share and discuss preliminary findings, inform future research and develop dissemination methods. The discussion of preliminary findings provides the opportunity to discuss findings within the context of pwMS and clinicians' experiences of adherence to physical rehabilitation delivered via tele-rehabilitation.

Patient and Public Involvement and Engagement:

Patient and Public Involvement and Engagement (PPIE) was sought in the development of the review's aims and research questions alongside the identification of possible dissemination methods to utilise following the completion of the review. The PPIE was undertaken with a group of four pwMS who provided written feedback on a lay summary of the scoping review protocol. Videoconferencing was then used with three pwMS from this group to explore further their priorities and experience of the use of tele-rehabilitation. This included discussions regarding their general experiences of tele-rehabilitation use alongside specific questions regarding adherence when therapeutic physical rehabilitation programmes have been delivered via tele-rehabilitation.

The feedback regarding the importance of the proposed area of research was useful in shaping the context of the review and its research questions. The PPIE feedback has been incorporated into the protocol through formulation of a research question relating to the experiences of pwMS and identification of dissemination methods as detailed below.

ETHICS AND DISSEMINATION:

Ethical approval was not required in the development of this protocol as the planned methodology involves the review of publicly available data.

The findings of the review will be submitted to a peer reviewed journal and for presentation at conferences. Further dissemination to clinicians working with pwMS will be guided by the consultation process. PPIE in the design of this protocol identified discussion with MS charities e.g. MS Trust regarding use of their social media or website platforms and newsletters to promote key findings as an important potential method of dissemination to pwMS, their families and carers. Further PPIE input through the consultation stage of the review may identify further appropriate dissemination methods for pwMS.

AUTHOR CONTRIBUTIONS:

G. Goldsmith, J. Bollen , J. Freeman, V. Salmon , S. Dean

For peer review only - http://bmjopen.bmj.com/site/about/guidelines.xhtml

Idea for scoping review (GG) with help from all authors in it's development. Refinement of aims and objectives by GG, JB, JF and SD. Search strategy written by GG with input from PenARC Information Specialist and all authors. Methods drafted by GG with input from all authors and PenARC search and review clinic team. All authors read and commented on the final draft.

ACKNOWLEDGEMENTS:

PPIE contributors: Thank you to Lynn Tatnell, Jessica Hole and Melanie Guy and other PPIE contributors for their time and help in reviewing the protocol and their contribution to it's development. Thank you to PenARC PPIE team for their help and support in arranging the PPIE contributions to this protocol.

FUNDING STATEMENT:

This work was supported by a Health Education England and the National Institute of Health (NIHR) Research South West Internship funding award (GG). SD's time is partially supported by the NIHR Applied Research Collaboration South West Peninsula. The views expressed in this publication are those of the author(s) and not necessarily those of the NIHR or the Department of Health and Social Care.

COMPETING INTEREST STATEMENT:

Geraldine Goldsmith received funding through a HEE/NIHR ICA Internship (Nov 2020 – April 2021).

DATA SHARING STATEMENT:

Data sharing not applicable as no datasets generated and/or analysed for this study.

FULL REFERENCES:

1. World Health Organisation Eduardo Sabaté (ed.) *Adherence to long term therapies: evidence for action* World Health Organisation (2003) Available from: https://apps.who.int/iris/bitstream/handle/10665/42682/9241545992.pdf?sequence=1&isAllowed=y (accessed 13 Dec 2021)

2 Neter E, Wolkowitz A, Glass-Marmor L, et al. Multiple modality approach to assess adherence to medications across time in Multiple Sclerosis. *Mult Scler Relat Disord*. 2020;May:40:101951. doi: 10.1016/j.msard.2020.101951. [published online 15 Jan 2020]

For peer review only - http://bmjopen.bmj.com/site/about/guidelines.xhtml

3 Quintana-Navarro GM, Alcala-Diaz JF, Lopez-Moreno J, et al. Long-term dietary adherence and changes in dietary intake in coronary patients after intervention with a Mediterranean diet or a low-fat diet: the CORDIOPREV randomized trial. *Eur J Nutr*. 2020;59(5):2099-2110. doi: 10.1007/s00394-019-02059-5. [published online 24 Jul 2019]

4 Holt CJ, McKay CD, Truong LK, et al. Sticking to It: A Scoping Review of Adherence to Exercise Therapy Interventions in Children and Adolescents With Musculoskeletal Conditions. *J Orthop Sports Phys Ther.* 2020;50(9):503-515. doi: 10.2519/jospt.2020.9715. [published online 1 Aug 2020]

5. Trueman, P; Taylor, DG; Lowson K, et al. *Evaluation of the Scale, Cause and Costs of Waste Medicines. Final Report*. York Health Economics Consortium, The School of Pharmacy, University of London (2010). Available from: https://discovery.ucl.ac.uk/id/eprint/1350234/1/Evaluation_of_NHS_Medicines_Waste__web_publication_version.pdf (accessed 13 Dec 2021)

6. Dennett R, Madsen LT, Connolly L, et al. Adherence and drop-out in randomized controlled trials of exercise interventions in people with multiple sclerosis: A systematic review and meta-analyses. *Mult Scler Relat Disord*. 2020;43:102169. doi: 10.1016/j.msard.2020.102169. [published online 19 May 2020]

7. Bailey DL, Holden MA, Foster NE, et al Defining adherence to therapeutic exercise for musculoskeletal pain: a systematic review *Br J Sports Med* 2020;54:326-331. doi: 10.1136/bjsports-2017-098742. [published online 6 June 2018]

8. World Health Organisation *WHO Guidelines on Physical Activity and Sedentary Behaviour* The World Health Organisation 2020 Available from: https://apps.who.int/iris/bitstream/handle/10665/336656/9789240015128-eng.pdf?sequence=1&isAllowed=y (accessed 21 Nov 2021)

9. Laver KE, Adey-Wakeling Z, Crotty M, et al. Telerehabilitation services for stroke. *Cochrane Database of Syst Rev*. 2020(1). doi: 10.1002/14651858.CD010255.pub3.

10. Di Tella S, Pagliari C, Blasi V, et al. Integrated telerehabilitation approach in multiple sclerosis: A systematic review and meta-analysis. *J Telemed Telecare*. 2020;26(7-8):385-399. doi: 10.1177/1357633X19850381. [published online 27 May 2019]

11. Department of Health and Social Care *UK Chief Medical Officers' Physical Activity Report* 2019 https://assets.publishing.service.gov.uk/government/uploads/system/uploads/attachment_data/file/832868/uk-chief-medical-officers-physical-activity-guidelines.pdf (accessed 21 Nov 2021)

12. Essery R, Geraghty AW, Kirby S, et al. Predictors of adherence to home-based physical therapies: a systematic review. *Disabil Rehabil*. 2017;39(6):519-534. doi: 10.3109/09638288.2016.1153160. [published online 21 April 2016]

For peer review only - http://bmjopen.bmj.com/site/about/guidelines.xhtml

13. Argent, R, Daly A, Caulfield B. Patient Involvement With Home-Based Exercise Programs: Can Connected Health Interventions Influence Adherence? *JMIR Mhealth Uhealth* 2018;6(3):e47. doi: **10.2196/mhealth.8518** [published online 1 Mar 2018]

14. Bollen JC, Dean SG, Siegert RJ, et al. A systematic review of measures of self-reported adherence to unsupervised home-based rehabilitation exercise programmes, and their psychometric properties. *BMJ open* 2014;4(6):e005044. doi: 10.1136/bmjopen-2014-005044

15. Casey B, Coote S, Hayes S, et al. Changing Physical Activity Behavior in People With Multiple Sclerosis: A Systematic Review and Meta-Analysis. *Arch Phys Med Rehabil*. 2018;99(10):2059-2075. doi: 10.1016/j.apmr.2017.12.013. [published online 11 Jan 2018]

16. Meade LB, Bearne LM, Sweeney LH, et al. Behaviour change techniques associated with adherence to prescribed exercise in patients with persistent musculoskeletal pain: Systematic review. *Br J Health Psychol*. 2019;24(1):10-30. doi: 10.1111/bjhp.12324. [published online 17 June 2018]

17. Willett M, Duda J, Fenton S, et al. Effectiveness of behaviour change techniques in physiotherapy interventions to promote physical activity adherence in lower limb osteoarthritis patients: A systematic review. *PLoS One*. 2019;14(7):e0219482. doi: 10.1371/journal.pone.0219482.

18. Michie, S., van Stralen, M.M., West, R. (2011) The behaviour change wheel: A new method for characterising and designing behaviour change interventions *Implement Sci.* 2011;6:42. doi: 10.1186/1748-5908-6-42

19. Coulter EH, Bond S, Dalgas U, et al. The effectiveness of interventions targeting physical activity and/or sedentary behaviour in people with Multiple Sclerosis: a systematic review. *Disabil Rehabil*. 2020;42(5):594-612. doi: 10.1080/09638288.2018.1503737. [published online 5 Oct 2018]

20. Sangelaji B, Smith CM, Paul L, et al. The effectiveness of behaviour change interventions to increase physical activity participation in people with multiple sclerosis: a systematic review and meta-analysis. *Clin Rehabil*. 2016;30(6):559-76. doi: 10.1177/0269215515595274. [published online 21 July 2015]

21. MS Society *What is MS?* Available from: https://www.mssociety.org.uk/about-ms/what-is-ms [accessed: 28 Dec 2020]

22. Rietberg MB, Brooks D, Uitdehaag BM, et al. Exercise therapy for multiple sclerosis. *Cochrane Database Syst Rev*.2005(1):CD003980. doi:10.1002/14651858.CD003980.pub2.

23. Amatya B, Khan F, Galea M. Rehabilitation for people with multiple sclerosis: an overview of Cochrane Reviews. *Cochrane Database of Syst Rev*. 2019(1). doi: 10.1002/14651858.CD12732.pub2

For peer review only - http://bmjopen.bmj.com/site/about/guidelines.xhtml

24. Stuifbergen AK, Blozis SA, Harrison TC, et al. Exercise, functional limitations, and quality of life: a longitudinal study of persons with multiple sclerosis. *Arch Phys Med Rehabil* 2006;87:935–943. doi: 10.1016/j.apmr.2006.04.003.

25. Motl RW, Sandroff BM, Kwakkel G, et al. Exercise in patients with multiple sclerosis. *Lancet neurol*. 2017;16(10):848-56. doi: 10.1016/S1474-4422(17)30281-8. [12 Sep 2017]

26. Shaw MT, Best P, Frontario A, et al. Telerehabilitation benefits patients with multiple sclerosis in an urban setting. *J Telemed Telecare*. 2021;27(1):39-45. doi: 10.1177/1357633X19861830. [published online 15 Jul 2019]

27. Rimmer JH, Thirumalai M, Young HJ, et al. Rationale and design of the tele-exercise and multiple sclerosis (TEAMS) study: A comparative effectiveness trial between a clinic- and home-based telerehabilitation intervention for adults with multiple sclerosis (MS) living in the deep south. *Contemp Clin Trials*. 2018;71:186-193. doi: 10.1016/j.cct.2018.05.016. [published online 30 May 2018]

28. Signal N, Martin T, Leys A, et al. Implementation of telerehabilitation in response to COVID-19: lessons learnt from neurorehabilitation clinical practice and education. *NZ J Physiother*2020;48:117-26. Available from: https://doi.org/10.15619/NZJP/48.3.03

29. Department of Health *NHS Long Term Plan* 2010 Available from: https://www.longtermplan.nhs.uk/online-version/chapter-5-digitally-enabled-care-will-go-mainstream-across-the-nhs/ (accessed 1 June 2020)

30. Pagliari C, Di Tella S, Jonsdottir J, et al. Effects of home-based virtual reality telerehabilitation system in people with multiple sclerosis: A randomized controlled trial. *J Telemed Telecare*. 2021;0(0) doi: 10.1177/1357633X211054839. [published online 1 Dec 2021]

31. Valenzuela T, Okubo Y, Woodbury A, et al. Adherence to Technology-Based Exercise Programs in Older Adults: A Systematic Review. *J Geriatr Phys Ther*. 2018;41(1):49-61. doi: 10.1519/JPT.0000000000000095

32. Paul L, Coulter EH, Miller L, et al. Web-based physiotherapy for people moderately affected with Multiple Sclerosis; quantitative and qualitative data from a randomized, controlled pilot study. *Clin Rehabil*. 2014;28(9):924-35. doi: 10.1177/0269215514527995. [published online 1 Apr 2014]

33. Paul L, Renfrew L, Freeman J, et al. Web-based physiotherapy for people affected by multiple sclerosis: a single blind, randomized controlled feasibility study. *Clin Rehabil*. 2019Mar;33(3):473-84. doi: 10.1177/0269215518817080. [published online 4 Dec 2018]

34. Dennett R, Coulter E, Paul L, et al. A qualitative exploration of the participants ' experience of a web-based physiotherapy program for people with multiple sclerosis: Does it impact on the ability to increase and sustain engagement in physical activity?. *Disabil*

For peer review only - http://bmjopen.bmj.com/site/about/guidelines.xhtml

Rehabil. 2020;8;42(21):3007-14. doi: 10.1080/09638288.2019.1582717. [published online 23 Mar 2019]

35. Dlugonski D, Motl RW, McAuley E. Increasing physical activity in multiple sclerosis: replicating Internet intervention effects using objective and self-report outcomes. *J Rehabil Res Dev*. 2011;48(9):1129-36. doi: 10.1682/jrrd.2010.09.0192.

36. Rintala A, Hakala S, Paltamaa J, et al. Effectiveness of technology-based distance physical rehabilitation interventions on physical activity and walking in multiple sclerosis: a systematic review and meta-analysis of randomized controlled trials. *Disabil Rehabil*. 2018;40(4):373-387. doi: 10.1080/09638288.2016.1260649. [published online 15 Dec 2016]

37. Dennett, R., Gunn, H., Freeman, J.A. Effectiveness of and User Experience With Web-Based Interventions in Increasing Physical Activity Levels in People With Multiple Sclerosis: A Systematic Review, *Phys Ther*, 2018;98(8)679-690 https://doi.org/10.1093/ptj/pzy060

38. Levac D, Colquhoun H, & O'Brien K.K. Scoping studies: advancing the methodology. *Implementation Sci* 2010;5,69. https://doi.org/10.1186/1748-5908-5-69

39. Arksey, H. & O'Malley, L. Scoping studies: towards a methodological framework *Int J Soc Res Methodol* 2005;8(1):19-32, doi: 10.1080/1364557032000119616

40. Tricco, AC, Lillie, E, Zarin, W, et al. PRISMA extension for scoping reviews (PRISMA-ScR): checklist and explanation. *Ann Intern Med*. 2018,169(7):467-473. doi: 10.7326/M18-0850. [published online 4 Sep 2018]

41. Lefebvre C, Glanville J, Briscoe S, et al. Chapter 4: Searching for and selecting studies. In: Higgins JPT, Thomas J, Chandler J, Cumpston M, Li T, Page MJ, Welch VA (eds). *Cochrane Handbook for Systematic Reviews of Interventions* version 6.1 (updated September 2020). Cochrane, 2020 Available from www.training.cochrane.org/handbook. (accessed 13 Dec 2021)

42. Peretti A, Amenta F, Tayebati SK, et al. Telerehabilitation: Review of the State-of-the-Art and Areas of Application. *JMIR Rehabil Assist Technol*. 2017;4(2):e7. doi: 10.2196/rehab.7511.

43. Plüddemann A, Aronson JK, Onakpoya I, et al. Redefining rapid reviews: a flexible framework for restricted systematic reviews. *BMJ Evid Based Med*. 2018;23(6):201-203. doi: 10.1136/bmjebm-2018-110990. [published online 27 June 2018].

44. Bussiek PV, De Poli C, Bevan G A scoping review protocol to map the evidence on interventions to prevent overweight and obesity in children *BMJOpen* 2018;8:e019311. doi: 10.1136/bmjopen-2017-019311

45. Parker M, Fang X, Self-Brown SR, et al. Establishing how social capital is studied in relation to cardiovascular disease and identifying gaps for future research-A scoping review protocol. *PLoS one*. 2021;8;16(4):e0249751. doi: 10.1371/journal.pone.0249751.

For peer review only - http://bmjopen.bmj.com/site/about/guidelines.xhtml

46. Herman C, Mayer K, Sarwal A. Scoping review of prevalence of neurologic comorbidities in patients hospitalized for COVID-19. *Neurology*. 2020;14;95(2):77-84. doi: 10.1212/WNL.0000000000009673. [published online 28 Apr 2020]

47. Critical Appraisal Skills Programme *Checklists* Available from: https://casp-uk.net/casp-tools-checklists/ (accessed 27.12.22)

48. Centre for Reviews and Dissemination *Systematic reviews. CRD's guidance for undertaking reviews in healthcare CRD* University of York. 2008 Available from https://www.york.ac.uk/media/crd/Systematic_Reviews.pdf (accessed 14.12.2020)

WORD COUNT: 3286

For peer review only

For peer review only - http://bmjopen.bmj.com/site/about/guidelines.xhtml

Draft search strategies:

Embase (Ovid)

1 Multiple Sclerosis.ti,ab. 134911
2 demyelinat*.ti,ab. 54974
3 multiple sclerosis/ or demyelinating disease/ 159457
4 1 or 2 or 3 199828
5 (tele* or virtual* or digital* or video or remote or web based or technolog* or online or mobile app* or software or phone or internet).ti,ab. 2217979
6 (exercise* or rehabilitat* or physiotherap* or physical therap*).ti,ab. 749164
7 (physical* adj activ*).ti,ab. 195167
8 6 or 7 892244
9 5 and 8 87670
10 telecare/ or telecommunication/ or teleconference/ or telehealth/ or telemedicine/ or teleconsultation/ or telemonitoring/ or telerehabilitation/ or video consultation/ 100269
11 telephone interview/ or telephone/ 60517
12 videoconferencing/ 7864
13 wireless communication/ 7098
14 information technology/ 12885
15 internet/ or web-based intervention/ 122028
16 mobile application/ or mobile health application/ 22434
17 9 or 10 or 11 or 12 or 13 or 14 or 15 or 16 376530
18 (exercise* or rehabilitat* or physiotherap* or physical therap*).ti,ab. 749164
19 (physical* adj activ*).ti,ab. 195167
20 exp physiotherapy/ 102325
21 exp rehabilitation/ 462483
22 exp physiotherapist/ 26174
23 exp exercise/ 407525
24 exp physical activity/ 511247
25 18 or 19 or 20 or 21 or 22 or 23 or 24 1599769
26 4 and 17 and 251453
27 limit 26 to yr="1998 -Current" 1445

HMIC Health Management Information Consortium (Ovid)

1 Multiple Sclerosis.ti,ab. 317
2 demyelinat*.ti,ab. 2
3 exp Multiple sclerosis/ 290
4 1 or 2 or 3 391
5 (tele* or virtual* or digital* or video or remote or web based or technolog* or online or mobile app* or software or phone or internet).ti,ab. 21838
6 (exercise* or rehabilitat* or physiotherap* or physical therap*).ti,ab. 9345
7 (physical* adj activ*).ti,ab. 2529
8 6 or 7 11439
9 5 and 8 921
10 telehealth/ or telemedicine/ or telecare/ 2220
11 Telephone/ or Telephone interviewing/ or Telephone advice/ 267
12 information technology/ 5014
13 Internet/ 1352
14 9 or 10 or 11 or 12 or 13 8984
15 (exercise* or rehabilitat* or physiotherap* or physical therap*).ti,ab. 9345
16 (physical* adj activ*).ti,ab. 2529
17 exp exercise/ or exp physical activity/ or exp exercise therapy/ 2964
18 exp Rehabilitation/ 2116
19 exp Physiotherapy/ 910

For peer review only

20	exp Physiotherapists/	363
21	15 or 16 or 17 or 18 or 19 or 20	13304
22	4 and 14 and 2	14
23	limit 22 to yr="1998 -Current"	4

Proquest thesis and dissertations

Set	Search	Results
S7	S1 AND S6 AND S4	328
S6	S2 OR S3	51,057
S4	(TI, AB (exercise*)) OR (TI,AB (rehabilitat*)) AND (TI,AB (physiotherap*)) AND (TI,AB (physical therap*)) AND (TI,AB (physical* NEAR/1 activ*))Limits applied	7,641
S3	(TI, AB (phone)) OR (TI,AB (internet))Limits applied	8,963
S2	(TI, AB (tele*)) OR (TI,AB (virtual)) OR (TI,AB (digital)) OR (TI,AB (video)) OR (TI,AB (remote)) OR (TI,AB ("web based")) OR (TI,AB (technolog*)) OR (TI,AB (online)) OR (TI,AB ("mobile app*")) OR (TI,AB (software))Limits applied	52,313
S1	(TI,AB (Multiple sclerosis)) OR (TI,AB (demyelinat*))Limits applied	2,074

Limits applied = search dates 1998 – current

All searches performed on 14 databases

CINAHL

S20	PY 1998-current	107
S19	S4 AND S15 AND S18	408
S18	S16 OR S17	565,738
S17	(MH "Exercise+") OR (MH "Rehabilitation+") OR (MH "Home Rehabilitation+") OR (MH "Physical Therapy+") OR (MH "Physical Therapists")	425,188
S16	TI exercise* OR AB exercise* OR TI rehabilitat* OR AB rehabilitat* OR TI physiotherap* OR AB physiotherap* OR TI "physicial therap*" OR AB "physical therap*" OR TI "physical* N activ*" OR AB "physical* N activ*"	269,339
S15	S9 OR S10 OR S11 OR S12 OR S13 OR S14	159,431
S14	(MH "Mobile Applications")	11,645
S13	(MH "Information Technology")	15,291
S12	(MH "Wireless Communications") OR (MH "Internet") OR (MH "Telephone") OR (MH "Internet-Based Intervention")	84,100
S11	(MH "Videoconferencing") OR (MH "Teleconferencing")	4,983
S10	(MH "Telerehabilitation") OR (MH "Telecommunications") OR (MH "Telehealth") OR (MH "Telemedicine") OR (MH "Remote Consultation")	33,195
S9	S7 AND S8	25,827
S8	S5 OR S6	521,765
S7	TI exercise* OR AB exercise* OR TI rehabilitat* OR AB rehabilitat* OR TI physiotherap* OR AB physiotherap* OR TI "physical therap*" OR AB "physical therap*" OR TI "physical* N activ*" OR AB "physical* N activ*"	273,823
S6	TI technolog* OR AB technolog* OR TI online OR AB online OR TI "mobile app*" OR AB "mobile app*" OR TI software OR AB software OR TI phone OR AB phone OR TI internet OR AB internet	369,239
S5	TI tele* OR AB tele* OR TI virtual* OR AB virtual* OR TI digital* OR AB digital* OR TI video OR AB video OR TI remote OR AB remote OR TI "web based" OR AB "web based"	232,047
S4	S1 OR S2 OR S3	30,771
S3	TI "multiple sclerosis" OR AB "multiple sclerosis" OR TI demyelinat* OR AB demyelinat*	23,546
S2	(MH "Demyelinating Diseases")	2,309
S1	(MH "Multiple Sclerosis")	22,249

Pedro:

Abstract & Title: "multiple sclerosis" tele*
Subdiscipline: neurology
Match all search terms (AND)

For peer review only

World Health Organisation International Clinical Trials Registry Platform portal:

- Multiple sclerosis AND tele* OR remote OR digital OR online OR phone OR internet OR virtual OR web OR software OR technolog* OR mobile OR video OR mobile
- Multiple sclerosis AND exercise* or rehabilitat* or physiotherap* or "physical therap*" or "physical activ*"

US National Library of Medicine Registry of Clinical Trials:

(telerehabilitation OR telemedicine OR telecare OR phone OR digital OR remote OR online OR mobile OR software OR video OR web OR techolog* OR internet) AND (exercise OR rehab* OR "physical activ*" OR "physical therapist") | Multiple Sclerosis

Cochrane central register of controlled trials Date Run: 08/12/2022 15:05:37

ID	Search	Hits
#1	MeSH descriptor: [Multiple Sclerosis] explode all trees	4016
#2	"multiple sclerosis":ti,ab	10983
#3	demyelinat*:ti,ab	1142
#4	#1 or #2 or #3	11891
#5	[mh telerehabilitation] or [mh telecommunications] or [mh telemedicine] or [mh "remote consultation"] or [mh telephone] or [mh "video conferencing"] or [mh "wireless technology"] or [mh internet] or [mh "information technology"] or [mh "mobile applications"]	12743
#6	(tele* or virtual* or video or remote* or "web based" or technolog* or online or "mobile app*" or software or phone or internet):ti,ab	137796
#7	(exercise* or rehabilitat* or physiotherap* or "physical therap*"):ti,ab	146032
#8	(physical* NEXT activ*):ti,ab	36900
#9	#7 or #8	166485
#10	#9 and #6	22937
#11	#10 or #5	33893
#12	[mh rehabilitation] or [mh exercise] or [mh "physical therapy modalities"] or [mh "physical therapy speciality"] or [mh "physical therapists"]	62360
#13	#12 or #9	191391
#14	#13 and #11 and #4 with Cochrane Library publication date from Jan 1998 to present	511

Websites:

The names of websites to be searched alongside search terms are provided below. On each website, authors will review the first three pages of search results. If there is no search function within a website, authors will look under relevant sections on that website, such as 'publications' or 'conferences'.

Websites to be searched:
MS Society, MS Trust, Chartered Society of Physiotherapy, UK Society of Behavioural Medicine, International Society of Behavioural Medicine, Open Grey, National MS Society, Rehabilitation in MS (RIMS), European Committee for Treatment and Research in MS.

Search terms:
Tele, virtual, multiple sclerosis, exercise

For peer review only

Adherence to physical rehabilitation delivered via tele-rehabilitation for people with Multiple Sclerosis: a scoping review protocol

Journal:	*BMJ Open*
Manuscript ID	bmjopen-2022-062548.R2
Article Type:	Protocol
Date Submitted by the Author:	17-Feb-2023
Complete List of Authors:	Goldsmith, Geraldine; University of Exeter<br>Bollen, Jessica; PenCLAHRC University of Exeter Medical School,<br>Salmon , Victoria ; University of Exeter,<br>Freeman, Jennifer; Peninsula Allied Hlth Ctr, Faculty of Health and Human Sciences<br>Dean, Sarah; PenCLAHRC University of Exeter Medical School,
**Primary Subject Heading**:	Rehabilitation medicine
Secondary Subject Heading:	Neurology
Keywords:	Multiple sclerosis < NEUROLOGY, Telemedicine < BIOTECHNOLOGY & BIOINFORMATICS, REHABILITATION MEDICINE

SCHOLARONE™
Manuscripts

Adherence to physical rehabilitation delivered via tele-rehabilitation for people with Multiple Sclerosis: a scoping review protocol

Corresponding author:
Geraldine Goldsmith
Exeter Community Neuro-rehabilitation Team, Poltimore Centre, Exeter Community Hospital, Hospital Lane, Exeter, EX1 3RB
g.goldsmith@exeter.ac.uk

Co-authors:

Dr Jessica Bollen
University of Exeter Medical School
Exeter, UK

Dr Victoria Salmon
University of Exeter Medical School
Exeter, UK

Professor Jennifer Freeman
Faculty of Health, School of Health Professions
University of Plymouth
Plymouth, UK

Professor Sarah Dean
University of Exeter Medical School
Exeter, UK

Word count: 3336

For peer review only - http://bmjopen.bmj.com/site/about/guidelines.xhtml

ABSTRACT:

Introduction: Using tele-rehabilitation methods to deliver exercise, physical activity (PA) and behaviour change interventions for people with Multiple Sclerosis (pwMS) has increased in recent years, especially since the SARS-CoV-2 pandemic. This scoping review aims to provide an overview of the literature regarding adherence to therapeutic exercise and PA delivered via tele-rehabilitation for pwMS.

Methods and analysis: Frameworks described by Arksey and O'Malley (2005) and Levac *et al* (2010) underpin the methods. The following databases will be searched from 1998 to present: Medline (Ovid), Embase (Ovid), Cinahl (EBSCOhost), Health Management Information Consortium Database, ProQuest Dissertations and Theses Global, Pedro, Cochrane central register of controlled trials, US National Library of Medicine Registry of Clinical Trials, World Health Organisation International Clinical Trials Registry Platform portal, and The Cochrane Database of Systematic Reviews. To identify papers not included in databases, relevant websites will be searched. Searches are planned for 2023. With the exception of study protocols, papers of any study design will be included. Papers reporting information regarding adherence in the context of prescribed therapeutic exercise and PA delivered via tele-rehabilitation for pwMS will be included. Information relating to adherence may comprise; methods of reporting adherence, adherence levels (e.g. exercise diaries, pedometers), investigation of pwMS 'and therapists 'experiences of adherence or a discussion of adherence. Eligibility criteria and a custom data extraction form will be piloted on a sample of papers. Quality assessment of included studies will use Critical Appraisal Skills Programme checklists. Data analysis will involve categorisation, enabling findings relating to study characteristics and research questions to be presented in narrative and tabular format.

Ethics and dissemination: Ethical approval was not required for this protocol. Findings will be submitted to a peer reviewed journal and presented at conferences. Consultation with pwMS and clinicians will help to identify other dissemination methods.

Protocol registration: www.osf.io

For peer review only - http://bmjopen.bmj.com/site/about/guidelines.xhtml

ARTICLE SUMMARY:

Strengths and limitations of this study:

- The SARS-CoV-2 pandemic has resulted in a rapid increase in research into tele-rehabilitation delivery of exercise and PA interventions, with this scoping review methodology well suited to the broad and emerging nature of this evidence.
- Decisions regarding study selection, data extraction and quality appraisal will be undertaken by one reviewer, however to reduce the risk of bias, a second reviewer will check 20% of these decisions.
- The inclusion of critical appraisal of studies will aid the interpretation of results and help to identify gaps within the research and the quality of the evidence base.
- The search strategy includes peer-reviewed evidence from electronic databases alongside organisation websites and conference proceedings.
- Patient and Public Involvement and Engagement was sought in the development of this scoping review, with a plan for further consultation with stakeholders during the review process.

INTRODUCTION:

Adherence is an important predictor of health outcomes [1] with higher adherence levels associated with improved treatment success across a range of healthcare interventions and patient populations [2-4]. Adherence to treatment regimes has the potential to impact healthcare costs; one striking example is where the NHS could save £500 million annually if medication adherence was improved in five key health conditions [5]. This example illustrates the same impact non-adherence may have on other treatment intervention costs, including those relevant to this review in exercise and physical activity (PA) programmes.

Adherence is often used interchangeably with terms such as compliance, participation, and concordance [6], despite the different meanings of these words [7]. The issue is complicated further within therapeutic exercise prescription where various parameters of adherence i.e. the aspects of adherence that can be measured, have been identified [7]. These parameters include the frequency of exercise (e.g. repetitions or sets), the quality of the exercises performed, but also attendance which is only a proxy marker of actually doing the exercises. These authors suggest that whilst several parameters may be relevant to therapeutic exercise, there is a lack of consensus regarding the relevance and importance in specific contexts [7].

For clarity and to encompass the multifaceted nature of the concept and parameters of adherence, within this paper the term adherence will be used, as defined by The World Health Organisation (WHO); “the extent to which a person's behaviour; taking medication, following a diet, and/or executing lifestyle changes, corresponds with agreed recommendations from a health care provider” [1](p.3). For clarity within this paper, the WHO’s definitions of exercise and PA will be utilised, with the term physical rehabilitation describing physical exercise and PA programmes prescribed with a therapeutic purpose. PA is defined by WHO as “any bodily movement produced by skeletal muscles that requires energy expenditure” [8](p.vii), and exercise as a subcategory of PA which is “planned,

structured, repetitive, purposeful in the sense that the improvement or maintenance of one or more components of physical fitness is the objective" [8](p.vi). Throughout this paper, the term tele-rehabilitation will refer to the range of technologies which are being used as the method of communication between the rehabilitation professional and patient [9], allowing for a double communication loop, whereby the patient's performance is monitored and relayed to the clinician who can then respond with appropriate feedback [10].

Although the beneficial effects of physical rehabilitation are well documented [11], adherence to such programmes remains problematic with adherence levels to home based unsupervised exercise reported to be as low as 30% [12]. As adherence is a key predictor of the effectiveness of exercise programmes [7], there is a clear need to improve adherence to physical rehabilitation programmes to maximise clinical outcomes. However without a gold standard way to measure adherence to unsupervised exercise [13] and a lack of validated measures [14], it can be difficult to interpret study results [4]; a poor outcome could be due to poor adherence or a genuine lack of intervention efficacy.

To improve patient adherence to physical rehabilitation, some studies have integrated behavioural change techniques (BCTs) into their programmes [15-17]. BCTs are the active ingredient, or unit of change, within behavioural interventions used to modify a specified behaviour [15, 18]. One typical BCT for physical rehabilitation would be setting an exercise behaviour goal [17]. The integration of BCTs into physical rehabilitation programmes has elicited positive results across clinical populations including people living with Multiple Sclerosis (pwMS) [15-17, 19, 20].

Multiple Sclerosis (MS) is a progressive condition of the central nervous system, affecting over 130,000 people in the UK [21]. Typically, people experience a myriad of sensory, cognitive and motor impairments with subsequent limitations in function [22, 23], which can impact negatively upon engagement in exercise and physical activity [24]. The benefits of exercise for improving symptoms, functional abilities, and quality of life for pwMS are well established [25] and physical rehabilitation programmes are frequently prescribed to pwMS [21]. However, pwMS are less physically active than the general population [25] and often experience difficulties in attending out-patient rehabilitation appointments [26], limiting their access to physical rehabilitation advice and prescription.

Tele-rehabilitation has been proposed as a potential solution for patients who have difficulty in attending out-patient rehabilitation services [27], and has been promoted within a wider rehabilitation context during the SARS-CoV-2 pandemic [28] with its use likely to continue in clinical practice in line with the Department of Health's Long Term Plan [29]. Within the context of rehabilitation, the 'double communication loop' is an essential element of the telerehabilitation as it allows clinicians to adjust or progress a patient's exercise programme according to their performance [10]. When delivering interventions via tele-rehabilitation, the interaction between clinician and patient may be synchronous (in real time), asynchronous (not in real time) or mixed, with the feedback received by the clinician delivered online (within the intervention) or offline (with a delay) [10, 30].

Within rehabilitation, technology can be utilised in various contexts including; to enable the communication between the patient and clinician e.g. video conferencing, or as the

For peer review only - http://bmjopen.bmj.com/site/about/guidelines.xhtml

rehabilitation intervention itself e.g. using wii fit games to improve upper limb motor function. Distinguishing between these two uses of technology is important when attempting to evaluate adherence to physical rehabilitation programmes, as using technology as the rehabilitation intervention may affect adherence [13, 31]. This review is interested in exploring whether adherence levels and the effectiveness of BCTs delivered via tele-rehabilitation, within the context of a physical rehabilitation programme, may differ to those delivered in face-to-face settings.

Studies investigating the use of tele-rehabilitation to deliver exercise programmes and interventions to increase PA for pwMS have sought participants' [32-35] and therapists' [33] views on the programmes, however the authors are not aware of any reviews that have provided an overview of these data. Difficulties with exercise progression were reported by therapists when their communication with participants was conducted via email and online exercise diaries as they were unable to observe participants perform their exercises [33]. Participants have found the tele-rehabilitation delivery acceptable [33, 35], and that it increased flexibility [32-34] as well as reducing the transport and physical energy costs of attending appointments [32]. Systematic reviews have investigated the use of tele-rehabilitation to deliver physical rehabilitation [36, 37], as well as the use of BCTs across tele-rehabilitation and in-person settings for pwMS [15, 19, 20]. However these reviews have either; focused on PA levels as the outcome of interest [15, 19, 20], did not explore adherence to the intervention [36], or included limited information detailing participants 'adherence to the prescribed physical rehabilitation programmes [37]. This scoping review aims to address this gap by specifically focusing on detailing the reported level of adherence to physical rehabilitation programmes, use of BCTs and the experiences of pwMS and therapists in adhering to these tele-rehabilitation delivered programmes.

METHODS AND ANALYSIS:

A scoping review methodology is particularly relevant in emerging fields [38] such as tele-rehabilitation; mapping the extent and nature of research and identifying research gaps [39]. The methods developed for this scoping review are based on the 6 stage framework described by Arksey and O'Malley [39] and Levac et al [38]. This protocol is reported in line with the Preferred Reporting Items for Systematic Reviews and Meta Analyses Extension for Scoping Reviews (PRISMA-ScR) [40].

Stage 1: Identifying the research question

This scoping review aims to provide an overview of the literature regarding adherence to physical rehabilitation delivered via tele-rehabilitation for pwMS. The specific research questions identified are:

- What levels of adherence are reported by studies prescribing physical rehabilitation delivered via tele-rehabilitation for pwMS?
- To what degree are valid and reliable measures of adherence used within studies?
- Is there evidence of integration of BCTs within the physical rehabilitation programmes prescribed, and how well is this reported?

For peer review only - http://bmjopen.bmj.com/site/about/guidelines.xhtml

- Is there any evidence that integrated BCTs have influenced levels of adherence to the prescribed physical rehabilitation programmes?
- What are the reported experiences of pwMS and physical rehabilitation prescribers regarding adherence to physical rehabilitation?

Stage 2: Identifying relevant studies

Scoping searches were utilised to identify free text and controlled subject heading terms for the population, intervention and outcome, and a draft search strategy for Medline (Ovid) was developed (strategy 1). As outcomes may not be reported within the title and abstract or picked up by the controlled vocabulary function [41], a further search strategy (strategy 2) without terms relating to outcomes (i.e. adherence) was devised with assistance from an information specialist who provided advice regarding free text terms, appropriate databases and supplementary searching. The two strategies were then compared for duplicates. All papers identified in search strategy 1, were also identified in search strategy 2, however search strategy 2 identified further relevant papers that were not identified by the first strategy. A search strategy without terms relating to outcomes will therefore be utilised within the scoping review to ensure that relevant papers are not missed. Search strategies are presented within the supplementary file.

The search strategies will be adapted for the following databases: Embase (Ovid), Cinahl (EBSCOhost), Health Management Information Consortium (HMIC) Database, ProQuest Dissertations and Theses Global (PQDT), Pedro, Cochrane central register of controlled trials, US National Library of Medicine Registry of Clinical Trials, World Health Organisation International Clinical Trials Registry Platform portal. Databases will be searched from 1998 to present day to reflect the start of the scientific publication of tele-rehabilitation studies [42].

To identify relevant papers not included in bibliographic databases, the following organisation and associated conference websites will be searched: MS Society, MS Trust, Chartered Society of Physiotherapy, UK Society of Behavioural Medicine, International Society of Behavioural Medicine, Open Grey, National MS Society, Rehabilitation in MS (RIMS) and European Committee for Treatment and Research in MS (ECTRIMS).

A search log will be completed to record searches including the source and dates covered, platform, date of search and number of records yielded. Following study selection, the reference lists of all included studies will be searched for additional relevant papers.

Stage 3: Study selection

Papers reporting information relating to adults (18+) with MS will be included. If mixed populations are included within a study, the study will be included if separate data are reported for adult participants with MS. Information on physical rehabilitation; physical exercises, or PA prescribed for a therapeutic purpose must be included, whether this relates to individual or group delivery, as part of a multifactorial rehabilitation programme or a single intervention. Papers describing tele-rehabilitation provision of the physical rehabilitation programme meeting Laver et al’s [9] and Di Tella et al’s [10] descriptions will be included,

For peer review only - http://bmjopen.bmj.com/site/about/guidelines.xhtml

whether the tele-rehabilitation is used as the sole programme delivery method or in combination with another method such as face-to-face.

The review will include papers reporting information relating to the concept of adherence as defined by the WHO [1]. Information relating to adherence may comprise; a method of reporting adherence, adherence levels (e.g. exercise diaries, pedometers, questionnaires, measurement scales), investigation of pwMS' or therapists' experiences of adherence or a discussion of adherence.

Study protocols will be excluded, with papers of all other study designs included. In order to minimise language bias, studies in all languages will be eligible with relevant studies translated to English where possible; any that we are unable to translate will be excluded from the review and reason for exclusion noted as language.

Search results will be downloaded into EndNote and duplicates removed. If there are multiple reports of the same study, these will be compared to ensure that adequate information is obtained and the study's results are only used once. The study selection process will be piloted independently by two team members on 25 studies to check the interpretation of the eligibility criteria and consistency of use. Following the piloting process, the eligibility criteria may be refined.

One reviewer will screen the titles and abstracts of papers, with 20% checked independently by a second reviewer. Papers appearing to meet the eligibility criteria and those where it is unclear from the title and abstract as to whether the criteria have been met, will have their full text screened by one reviewer, with 20% screened by a second reviewer. Discrepancies between the two reviewers at any stage of the screening process will be resolved via consensus and discussion with a third person. These processes meet the requirements set out by Pluddemann *et al* [43] for the study selection stage of restricted systematic reviews and reflect the method used by other authors [44].

The number of studies excluded at both stages of screening alongside reasons for exclusion will be recorded to enable completion of a PRISMA flow diagram and narrative summary of the screening decision process.

Stage 4: Charting the data

Data extraction will be piloted on five included studies independently by two reviewers, with processes amended as required. This piloting process will ensure that the instructions are applied consistently and the planned data extraction allows relevant characteristics of each study to be presented in the review alongside a quality assessment and details relating to the research questions and review aim.

Following the methodology in other scoping reviews and protocols [45, 46], and meeting the minimum requirements for restricted systematic reviews as set out by Pluddemann et al [43], data will be extracted into a custom data extraction form by one reviewer. To reduce data bias error, partial verification (20%) of data extraction will be undertaken by a second reviewer [43]. Disagreements will be resolved by consensus and discussion with a third

For peer review only - http://bmjopen.bmj.com/site/about/guidelines.xhtml

reviewer. The date of extraction and reviewer undertaking the extraction will be recorded alongside key information regarding the paper including: Author; year and country of publication; aim and objectives of paper; participant / population information; Methods (including study design, blinding, randomisation, time points of data collection); Intervention description (including details of tele-rehabilitation methods; synchronous/asynchronous delivery, online/offline feedback, type of technology, exercise or PA interventions, BCTs, comparators); Outcomes and results (including adherence measurement tools e.g. session attendance or exercise diaries, information relating to participants 'experiences); key findings and discussion points relating to the research questions. Where relevant information is missing from articles, authors will be contacted for further information where possible.

Stage 5: Collating, summarising and reporting the results

This stage will involve three distinct steps; analysis of the data, reporting of the results and outcome in relation to the research questions; and consideration of the meaning of the findings [38]. The methodological quality of included studies will be assessed independently by one reviewer with 20% of decisions checked by a second reviewer, both using tools from the Critical Appraisal Skills Programme suite [47]. A third reviewer will be available to mediate any disagreements.

Analysis of the data:
The planned analysis of data will occur in two stages. Firstly an analysis of the data extracted from all included studies with regard to their characteristics and quality appraisal will be undertaken. Secondly the research questions will be used to structure and organise an analysis of study findings, outcomes and interventions. Where appropriate, frequency counts will be utilised for information including study design, use of adherence measures (e.g. exercise diary), reporting of adherence levels and intervention characteristics (e.g. type of tele-rehabilitation and BCTs utilised). Data relating to participant adherence levels will be analysed through frequency counts to identify the number of studies reporting a level of adherence and using each measurement tool. Data relating to participants' and prescribers' experiences of adherence within studies will be analysed through the use of categories and themes.

Reporting of the results and outcome in relation to the research questions:
The characteristics of all included studies incorporating study design, population, interventions and quality appraisal will be presented in a table and narrative summary. Extracted data and the review's findings will be presented in a narrative summary addressing each of the research questions, with the use of tables and diagrams to aid interpretation where appropriate. Data relating to the level of adherence described by studies (research question 1) will be reported with reference to the measurement tool used (research question 2) and adherence parameter measured to provide greater context and appropriate grouping of data relating to adherence levels.

Consideration of the meaning of the findings:
Findings will be summarised in the context of whether the review's aims have been met and research questions answered. Discussion of the key findings will be included alongside identification of gaps in current knowledge and corresponding implications for future

For peer review only - http://bmjopen.bmj.com/site/about/guidelines.xhtml

research. The quality assessment of papers may aid the interpretation of results [48] and identification of gaps within the literature [38].

Stage 6: Consultation

It is planned that consultation will involve pwMS and therapists working with pwMS in community settings (those working with pwMS in their own homes and out-patient settings). The aims of the planned consultation are to share and discuss preliminary findings, inform future research and develop dissemination methods. The discussion of preliminary findings provides the opportunity to discuss findings within the context of pwMS and clinicians' experiences of adherence to physical rehabilitation delivered via tele-rehabilitation.

Patient and Public Involvement and Engagement:

Patient and Public Involvement and Engagement (PPIE) was sought in the development of the review's aims and research questions alongside the identification of possible dissemination methods to utilise following the completion of the review. The PPIE was undertaken with a group of four pwMS who provided written feedback on a lay summary of the scoping review protocol. Videoconferencing was then used with three pwMS from this group to explore further their priorities and experience of the use of tele-rehabilitation. This included discussions regarding their general experiences of tele-rehabilitation use alongside specific questions regarding adherence when therapeutic physical rehabilitation programmes have been delivered via tele-rehabilitation.

The feedback regarding the importance of the proposed area of research was useful in shaping the context of the review and its research questions. The PPIE feedback has been incorporated into the protocol through formulation of a research question relating to the experiences of pwMS and identification of dissemination methods as detailed below.

ETHICS AND DISSEMINATION:

Ethical approval was not required in the development of this protocol as the planned methodology involves the review of publicly available data.

The findings of the review will be submitted to a peer reviewed journal and for presentation at conferences. Further dissemination to clinicians working with pwMS will be guided by the consultation process. PPIE in the design of this protocol identified discussion with MS charities e.g. MS Trust regarding use of their social media or website platforms and newsletters to promote key findings as an important potential method of dissemination to pwMS, their families and carers. Further PPIE input through the consultation stage of the review may identify further appropriate dissemination methods for pwMS.

AUTHOR CONTRIBUTIONS:

G. Goldsmith, J. Bollen , J. Freeman, V. Salmon , S. Dean

For peer review only - http://bmjopen.bmj.com/site/about/guidelines.xhtml

Idea for scoping review (GG) with help from JB, VS, JF and SD in it's development. Refinement of aims and objectives by GG, JB, JF and SD. Search strategy written by GG with input from PenARC Information Specialist, JB, VS, JF and SD. Methods drafted by GG with input from JB, VS, JF, SD and PenARC search and review clinic team. All authors read and commented on the final draft.

ACKNOWLEDGEMENTS:

PPIE contributors: Thank you to Lynn Tatnell, Jessica Hole and Melanie Guy and other PPIE contributors for their time and help in reviewing the protocol and their contribution to it's development. Thank you to PenARC PPIE team for their help and support in arranging the PPIE contributions to this protocol.

FUNDING STATEMENT:

This work was supported by a Health Education England and the National Institute of Health (NIHR) Research South West Internship funding award (GG). SD's time is partially supported by the NIHR Applied Research Collaboration South West Peninsula. The views expressed in this publication are those of the author(s) and not necessarily those of the NIHR or the Department of Health and Social Care.

COMPETING INTEREST STATEMENT:

Geraldine Goldsmith received funding through a HEE/NIHR ICA Internship (Nov 2020 – April 2021).

DATA SHARING STATEMENT:

Data sharing not applicable as no datasets generated and/or analysed for this study.

FULL REFERENCES:

1. World Health Organisation Eduardo Sabaté (ed.) *Adherence to long term therapies: evidence for action* World Health Organisation (2003) Available from: https://apps.who.int/iris/bitstream/handle/10665/42682/9241545992.pdf?sequence=1&isAllowed=y (accessed 13 Dec 2021)

2 Neter E, Wolkowitz A, Glass-Marmor L, et al. Multiple modality approach to assess adherence to medications across time in Multiple Sclerosis. *Mult Scler Relat Disord*. 2020;May:40:101951. doi: 10.1016/j.msard.2020.101951. [published online 15 Jan 2020]

For peer review only - http://bmjopen.bmj.com/site/about/guidelines.xhtml

3 Quintana-Navarro GM, Alcala-Diaz JF, Lopez-Moreno J, et al. Long-term dietary adherence and changes in dietary intake in coronary patients after intervention with a Mediterranean diet or a low-fat diet: the CORDIOPREV randomized trial. *Eur J Nutr*. 2020;59(5):2099-2110. doi: 10.1007/s00394-019-02059-5. [published online 24 Jul 2019]

4 Holt CJ, McKay CD, Truong LK, et al. Sticking to It: A Scoping Review of Adherence to Exercise Therapy Interventions in Children and Adolescents With Musculoskeletal Conditions. *J Orthop Sports Phys Ther.* 2020;50(9):503-515. doi: 10.2519/jospt.2020.9715. [published online 1 Aug 2020]

5. Trueman, P; Taylor, DG; Lowson K, et al. *Evaluation of the Scale, Cause and Costs of Waste Medicines. Final Report*. York Health Economics Consortium, The School of Pharmacy, University of London (2010). Available from: https://discovery.ucl.ac.uk/id/eprint/1350234/1/Evaluation_of_NHS_Medicines_Waste__web_publication_version.pdf (accessed 13 Dec 2021)

6. Dennett R, Madsen LT, Connolly L, et al. Adherence and drop-out in randomized controlled trials of exercise interventions in people with multiple sclerosis: A systematic review and meta-analyses. *Mult Scler Relat Disord*. 2020;43:102169. doi: 10.1016/j.msard.2020.102169. [published online 19 May 2020]

7. Bailey DL, Holden MA, Foster NE, et al Defining adherence to therapeutic exercise for musculoskeletal pain: a systematic review *Br J Sports Med* 2020;54:326-331. doi: 10.1136/bjsports-2017-098742. [published online 6 June 2018]

8. World Health Organisation *WHO Guidelines on Physical Activity and Sedentary Behaviour* The World Health Organisation 2020 Available from: https://apps.who.int/iris/bitstream/handle/10665/336656/9789240015128-eng.pdf?sequence=1&isAllowed=y (accessed 21 Nov 2021)

9. Laver KE, Adey-Wakeling Z, Crotty M, et al. Telerehabilitation services for stroke. *Cochrane Database of Syst Rev*. 2020(1). doi: 10.1002/14651858.CD010255.pub3.

10. Di Tella S, Pagliari C, Blasi V, et al. Integrated telerehabilitation approach in multiple sclerosis: A systematic review and meta-analysis. *J Telemed Telecare*. 2020;26(7-8):385-399. doi: 10.1177/1357633X19850381. [published online 27 May 2019]

11. Department of Health and Social Care *UK Chief Medical Officers' Physical Activity Report* 2019 https://assets.publishing.service.gov.uk/government/uploads/system/uploads/attachment_data/file/832868/uk-chief-medical-officers-physical-activity-guidelines.pdf (accessed 21 Nov 2021)

12. Essery R, Geraghty AW, Kirby S, et al. Predictors of adherence to home-based physical therapies: a systematic review. *Disabil Rehabil*. 2017;39(6):519-534. doi: 10.3109/09638288.2016.1153160. [published online 21 April 2016]

For peer review only - http://bmjopen.bmj.com/site/about/guidelines.xhtml

13. Argent, R, Daly A, Caulfield B. Patient Involvement With Home-Based Exercise Programs: Can Connected Health Interventions Influence Adherence? *JMIR Mhealth Uhealth* 2018;6(3):e47. doi: **10.2196/mhealth.8518** [published online 1 Mar 2018]

14. Bollen JC, Dean SG, Siegert RJ, et al. A systematic review of measures of self-reported adherence to unsupervised home-based rehabilitation exercise programmes, and their psychometric properties. *BMJ open* 2014;4(6):e005044. doi: 10.1136/bmjopen-2014-005044

15. Casey B, Coote S, Hayes S, et al. Changing Physical Activity Behavior in People With Multiple Sclerosis: A Systematic Review and Meta-Analysis. *Arch Phys Med Rehabil*. 2018;99(10):2059-2075. doi: 10.1016/j.apmr.2017.12.013. [published online 11 Jan 2018]

16. Meade LB, Bearne LM, Sweeney LH, et al. Behaviour change techniques associated with adherence to prescribed exercise in patients with persistent musculoskeletal pain: Systematic review. *Br J Health Psychol*. 2019;24(1):10-30. doi: 10.1111/bjhp.12324. [published online 17 June 2018]

17. Willett M, Duda J, Fenton S, et al. Effectiveness of behaviour change techniques in physiotherapy interventions to promote physical activity adherence in lower limb osteoarthritis patients: A systematic review. *PLoS One*. 2019;14(7):e0219482. doi: 10.1371/journal.pone.0219482.

18. Michie, S., van Stralen, M.M., West, R. (2011) The behaviour change wheel: A new method for characterising and designing behaviour change interventions *Implement Sci.* 2011;6:42. doi: 10.1186/1748-5908-6-42

19. Coulter EH, Bond S, Dalgas U, et al. The effectiveness of interventions targeting physical activity and/or sedentary behaviour in people with Multiple Sclerosis: a systematic review. *Disabil Rehabil*. 2020;42(5):594-612. doi: 10.1080/09638288.2018.1503737. [published online 5 Oct 2018]

20. Sangelaji B, Smith CM, Paul L, et al. The effectiveness of behaviour change interventions to increase physical activity participation in people with multiple sclerosis: a systematic review and meta-analysis. *Clin Rehabil*. 2016;30(6):559-76. doi: 10.1177/0269215515595274. [published online 21 July 2015]

21. MS Society *What is MS?* Available from: https://www.mssociety.org.uk/about-ms/what-is-ms [accessed: 28 Dec 2020]

22. Rietberg MB, Brooks D, Uitdehaag BM, et al. Exercise therapy for multiple sclerosis. *Cochrane Database Syst Rev*.2005(1):CD003980. doi:10.1002/14651858.CD003980.pub2.

23. Amatya B, Khan F, Galea M. Rehabilitation for people with multiple sclerosis: an overview of Cochrane Reviews. *Cochrane Database of Syst Rev*. 2019(1). doi: 10.1002/14651858.CD12732.pub2

For peer review only - http://bmjopen.bmj.com/site/about/guidelines.xhtml

24. Stuifbergen AK, Blozis SA, Harrison TC, et al. Exercise, functional limitations, and quality of life: a longitudinal study of persons with multiple sclerosis. *Arch Phys Med Rehabil* 2006;87:935–943. doi: 10.1016/j.apmr.2006.04.003.

25. Motl RW, Sandroff BM, Kwakkel G, et al. Exercise in patients with multiple sclerosis. *Lancet neurol*. 2017;16(10):848-56. doi: 10.1016/S1474-4422(17)30281-8. [12 Sep 2017]

26. Shaw MT, Best P, Frontario A, et al. Telerehabilitation benefits patients with multiple sclerosis in an urban setting. *J Telemed Telecare*. 2021;27(1):39-45. doi: 10.1177/1357633X19861830. [published online 15 Jul 2019]

27. Rimmer JH, Thirumalai M, Young HJ, et al. Rationale and design of the tele-exercise and multiple sclerosis (TEAMS) study: A comparative effectiveness trial between a clinic- and home-based telerehabilitation intervention for adults with multiple sclerosis (MS) living in the deep south. *Contemp Clin Trials*. 2018;71:186-193. doi: 10.1016/j.cct.2018.05.016. [published online 30 May 2018]

28. Signal N, Martin T, Leys A, et al. Implementation of telerehabilitation in response to COVID-19: lessons learnt from neurorehabilitation clinical practice and education. *NZ J Physiother*2020;48:117-26. Available from: https://doi.org/10.15619/NZJP/48.3.03

29. Department of Health *NHS Long Term Plan* 2010 Available from: https://www.longtermplan.nhs.uk/online-version/chapter-5-digitally-enabled-care-will-go-mainstream-across-the-nhs/ (accessed 1 June 2020)

30. Pagliari C, Di Tella S, Jonsdottir J, et al. Effects of home-based virtual reality telerehabilitation system in people with multiple sclerosis: A randomized controlled trial. *J Telemed Telecare*. 2021;0(0) doi: 10.1177/1357633X211054839. [published online 1 Dec 2021]

31. Valenzuela T, Okubo Y, Woodbury A, et al. Adherence to Technology-Based Exercise Programs in Older Adults: A Systematic Review. *J Geriatr Phys Ther*. 2018;41(1):49-61. doi: 10.1519/JPT.0000000000000095

32. Paul L, Coulter EH, Miller L, et al. Web-based physiotherapy for people moderately affected with Multiple Sclerosis; quantitative and qualitative data from a randomized, controlled pilot study. *Clin Rehabil*. 2014;28(9):924-35. doi: 10.1177/0269215514527995. [published online 1 Apr 2014]

33. Paul L, Renfrew L, Freeman J, et al. Web-based physiotherapy for people affected by multiple sclerosis: a single blind, randomized controlled feasibility study. *Clin Rehabil*. 2019Mar;33(3):473-84. doi: 10.1177/0269215518817080. [published online 4 Dec 2018]

34. Dennett R, Coulter E, Paul L, et al. A qualitative exploration of the participants ' experience of a web-based physiotherapy program for people with multiple sclerosis: Does it impact on the ability to increase and sustain engagement in physical activity?. *Disabil*

For peer review only - http://bmjopen.bmj.com/site/about/guidelines.xhtml

Rehabil. 2020;8;42(21):3007-14. doi: 10.1080/09638288.2019.1582717. [published online 23 Mar 2019]

35. Dlugonski D, Motl RW, McAuley E. Increasing physical activity in multiple sclerosis: replicating Internet intervention effects using objective and self-report outcomes. *J Rehabil Res Dev*. 2011;48(9):1129-36. doi: 10.1682/jrrd.2010.09.0192.

36. Rintala A, Hakala S, Paltamaa J, et al. Effectiveness of technology-based distance physical rehabilitation interventions on physical activity and walking in multiple sclerosis: a systematic review and meta-analysis of randomized controlled trials. *Disabil Rehabil*. 2018;40(4):373-387. doi: 10.1080/09638288.2016.1260649. [published online 15 Dec 2016]

37. Dennett, R., Gunn, H., Freeman, J.A. Effectiveness of and User Experience With Web-Based Interventions in Increasing Physical Activity Levels in People With Multiple Sclerosis: A Systematic Review, *Phys Ther*, 2018;98(8)679-690 https://doi.org/10.1093/ptj/pzy060

38. Levac D, Colquhoun H, & O'Brien K.K. Scoping studies: advancing the methodology. *Implementation Sci* 2010;5,69. https://doi.org/10.1186/1748-5908-5-69

39. Arksey, H. & O'Malley, L. Scoping studies: towards a methodological framework *Int J Soc Res Methodol* 2005;8(1):19-32, doi: 10.1080/1364557032000119616

40. Tricco, AC, Lillie, E, Zarin, W, et al. PRISMA extension for scoping reviews (PRISMA-ScR): checklist and explanation. *Ann Intern Med*. 2018,169(7):467-473. doi: 10.7326/M18-0850. [published online 4 Sep 2018]

41. Lefebvre C, Glanville J, Briscoe S, et al. Chapter 4: Searching for and selecting studies. In: Higgins JPT, Thomas J, Chandler J, Cumpston M, Li T, Page MJ, Welch VA (eds). *Cochrane Handbook for Systematic Reviews of Interventions* version 6.1 (updated September 2020). Cochrane, 2020 Available from www.training.cochrane.org/handbook. (accessed 13 Dec 2021)

42. Peretti A, Amenta F, Tayebati SK, et al. Telerehabilitation: Review of the State-of-the-Art and Areas of Application. *JMIR Rehabil Assist Technol*. 2017;4(2):e7. doi: 10.2196/rehab.7511.

43. Plüddemann A, Aronson JK, Onakpoya I, et al. Redefining rapid reviews: a flexible framework for restricted systematic reviews. *BMJ Evid Based Med*. 2018;23(6):201-203. doi: 10.1136/bmjebm-2018-110990. [published online 27 June 2018].

44. Bussiek PV, De Poli C, Bevan G A scoping review protocol to map the evidence on interventions to prevent overweight and obesity in children *BMJOpen* 2018;8:e019311. doi: 10.1136/bmjopen-2017-019311

45. Parker M, Fang X, Self-Brown SR, et al. Establishing how social capital is studied in relation to cardiovascular disease and identifying gaps for future research-A scoping review protocol. *PLoS one*. 2021;8;16(4):e0249751. doi: 10.1371/journal.pone.0249751.

For peer review only - http://bmjopen.bmj.com/site/about/guidelines.xhtml

46. Herman C, Mayer K, Sarwal A. Scoping review of prevalence of neurologic comorbidities in patients hospitalized for COVID-19. *Neurology*. 2020;14;95(2):77-84. doi: 10.1212/WNL.0000000000009673. [published online 28 Apr 2020]

47. Critical Appraisal Skills Programme *Checklists* Available from: https://casp-uk.net/casp-tools-checklists/ (accessed 27.12.22)

48. Centre for Reviews and Dissemination *Systematic reviews. CRD's guidance for undertaking reviews in healthcare CRD* University of York. 2008 Available from https://www.york.ac.uk/media/crd/Systematic_Reviews.pdf (accessed 14.12.2020)

WORD COUNT: 3336

For peer review only

For peer review only - http://bmjopen.bmj.com/site/about/guidelines.xhtml

Supplementary file

Medline (Ovid):

1 Multiple sclerosis.ti,ab. 85317
2 demyelinat*.ti,ab. 34867
3 multiple sclerosis/ or demyelinating diseases/ 70724
4 1 or 2 or 3 118843
5 (tele* or virtual* or digital* or video or remote or web based or technolog* or online or mobile app* or software or phone or internet).ti,ab. 1620083
6 (exercise* or rehabilitat* or physiotherap* or physical therap*).ti,ab. 541966
7 (physical* adj activ*).ti,ab. 142680
8 6 or 7 649188
9 5 and 8 56897
10 telecommunications/ or telemedicine/ or remote consultation/ or telerehabilitation/ or telephone/ or videoconferencing/ or wireless technology/ 61250
11 Information Technology/ 717
12 internet/ or internet-based intervention/ 80763
13 Mobile Applications/ 10527
14 9 or 10 or 11 or 12 or 13 195458
15 (exercise* or rehabilitat* or physiotherap* or physical therap*).ti,ab. 541966
16 (physical* adj activ*).ti,ab. 142680
17 exp Physical Therapy Modalities/ 173345
18 exp Rehabilitation/ 343384
19 exp Physical Therapists/2853
20 exp Exercise/ 236155
21 15 or 16 or 17 or 18 or 19 or 20 953695
22 4 and 14 and 21696
23 limit 22 to yr="1998 -Current" 690

For peer review only

Draft search strategies:

Embase (Ovid)

1 Multiple Sclerosis.ti,ab. 134911
2 demyelinat*.ti,ab. 54974
3 multiple sclerosis/ or demyelinating disease/ 159457
4 1 or 2 or 3 199828
5 (tele* or virtual* or digital* or video or remote or web based or technolog* or online or mobile app* or software or phone or internet).ti,ab. 2217979
6 (exercise* or rehabilitat* or physiotherap* or physical therap*).ti,ab. 749164
7 (physical* adj activ*).ti,ab. 195167
8 6 or 7 892244
9 5 and 8 87670
10 telecare/ or telecommunication/ or teleconference/ or telehealth/ or telemedicine/ or teleconsultation/ or telemonitoring/ or telerehabilitation/ or video consultation/ 100269
11 telephone interview/ or telephone/ 60517
12 videoconferencing/ 7864
13 wireless communication/ 7098
14 information technology/ 12885
15 internet/ or web-based intervention/ 122028
16 mobile application/ or mobile health application/ 22434
17 9 or 10 or 11 or 12 or 13 or 14 or 15 or 16 376530
18 (exercise* or rehabilitat* or physiotherap* or physical therap*).ti,ab. 749164
19 (physical* adj activ*).ti,ab. 195167
20 exp physiotherapy/ 102325
21 exp rehabilitation/ 462483
22 exp physiotherapist/ 26174
23 exp exercise/ 407525
24 exp physical activity/ 511247
25 18 or 19 or 20 or 21 or 22 or 23 or 24 1599769
26 4 and 17 and 251453
27 limit 26 to yr="1998 -Current" 1445

HMIC Health Management Information Consortium (Ovid)

1 Multiple Sclerosis.ti,ab. 317
2 demyelinat*.ti,ab. 2
3 exp Multiple sclerosis/ 290
4 1 or 2 or 3 391
5 (tele* or virtual* or digital* or video or remote or web based or technolog* or online or mobile app* or software or phone or internet).ti,ab. 21838
6 (exercise* or rehabilitat* or physiotherap* or physical therap*).ti,ab. 9345
7 (physical* adj activ*).ti,ab. 2529
8 6 or 7 11439
9 5 and 8 921
10 telehealth/ or telemedicine/ or telecare/ 2220
11 Telephone/ or Telephone interviewing/ or Telephone advice/ 267
12 information technology/ 5014
13 Internet/ 1352
14 9 or 10 or 11 or 12 or 13 8984
15 (exercise* or rehabilitat* or physiotherap* or physical therap*).ti,ab. 9345
16 (physical* adj activ*).ti,ab. 2529
17 exp exercise/ or exp physical activity/ or exp exercise therapy/ 2964
18 exp Rehabilitation/ 2116
19 exp Physiotherapy/ 910

For peer review only

20 exp Physiotherapists/ 363
21 15 or 16 or 17 or 18 or 19 or 20 13304
22 4 and 14 and 214
23 limit 22 to yr="1998 -Current" 4

Proquest thesis and dissertations

Set	Search	Results
S7	S1 AND S6 AND S4	328
S6	S2 OR S3	51,057
S4	(TI, AB (exercise*)) OR (TI,AB (rehabilitat*)) AND (TI,AB (physiotherap*)) AND (TI,AB (physical therap*)) AND (TI,AB (physical* NEAR/1 activ*))Limits applied	7,641
S3	(TI, AB (phone)) OR (TI,AB (internet))Limits applied	8,963
S2	(TI, AB (tele*)) OR (TI,AB (virtual)) OR (TI,AB (digital)) OR (TI,AB (video)) OR (TI,AB (remote)) OR (TI,AB ("web based")) OR (TI,AB (technolog*)) OR (TI,AB (online)) OR (TI,AB ("mobile app*")) OR (TI,AB (software))Limits applied	52,313
S1	(TI,AB (Multiple sclerosis)) OR (TI,AB (demyelinat*))Limits applied	2,074

Limits applied = search dates 1998 – current

All searches performed on 14 databases

CINAHL

S20	PY 1998-current	107
S19	S4 AND S15 AND S18	408
S18	S16 OR S17	565,738
S17	(MH "Exercise+") OR (MH "Rehabilitation+") OR (MH "Home Rehabilitation+") OR (MH "Physical Therapy+") OR (MH "Physical Therapists")	425,188
S16	TI exercise* OR AB exercise* OR TI rehabilitat* OR AB rehabilitat* OR TI physiotherap* OR AB physiotherap* OR TI "physicial therap*" OR AB "physical therap*" OR TI "physical* N activ*" OR AB "physical* N activ*"	269,339
S15	S9 OR S10 OR S11 OR S12 OR S13 OR S14	159,431
S14	(MH "Mobile Applications")	11,645
S13	(MH "Information Technology")	15,291
S12	(MH "Wireless Communications") OR (MH "Internet") OR (MH "Telephone") OR (MH "Internet-Based Intervention")	84,100
S11	(MH "Videoconferencing") OR (MH "Teleconferencing")	4,983
S10	(MH "Telerehabilitation") OR (MH "Telecommunications") OR (MH "Telehealth") OR (MH "Telemedicine") OR (MH "Remote Consultation")	33,195
S9	S7 AND S8	25,827
S8	S5 OR S6	521,765
S7	TI exercise* OR AB exercise* OR TI rehabilitat* OR AB rehabilitat* OR TI physiotherap* OR AB physiotherap* OR TI "physical therap*" OR AB "physical therap*" OR TI "physical* N activ*" OR AB "physical* N activ*"	273,823
S6	TI technolog* OR AB technolog* OR TI online OR AB online OR TI "mobile app*" OR AB "mobile app*" OR TI software OR AB software OR TI phone OR AB phone OR TI internet OR AB internet	369,239
S5	TI tele* OR AB tele* OR TI virtual* OR AB virtual* OR TI digital* OR AB digital* OR TI video OR AB video OR TI remote OR AB remote OR TI "web based" OR AB "web based"	232,047
S4	S1 OR S2 OR S3	30,771
S3	TI "multiple sclerosis" OR AB "multiple sclerosis" OR TI demyelinat* OR AB demyelinat*	23,546
S2	(MH "Demyelinating Diseases")	2,309
S1	(MH "Multiple Sclerosis")	22,249

Pedro:

Abstract & Title: "multiple sclerosis" tele*
Subdiscipline: neurology
Match all search terms (AND)

For peer review only

World Health Organisation International Clinical Trials Registry Platform portal:

- Multiple sclerosis AND tele* OR remote OR digital OR online OR phone OR internet OR virtual OR web OR software OR technolog* OR mobile OR video OR mobile
- Multiple sclerosis AND exercise* or rehabilitat* or physiotherap* or "physical therap*" or "physical activ*"

US National Library of Medicine Registry of Clinical Trials:

(telerehabilitation OR telemedicine OR telecare OR phone OR digital OR remote OR online OR mobile OR software OR video OR web OR techolog* OR internet) AND (exercise OR rehab* OR "physical activ*" OR "physical therapist") | Multiple Sclerosis

Cochrane central register of controlled trials Date Run: 08/12/2022 15:05:37

ID	Search	Hits
#1	MeSH descriptor: [Multiple Sclerosis] explode all trees	4016
#2	"multiple sclerosis":ti,ab	10983
#3	demyelinat*:ti,ab	1142
#4	#1 or #2 or #3	11891
#5	[mh telerehabilitation] or [mh telecommunications] or [mh telemedicine] or [mh "remote consultation"] or [mh telephone] or [mh "video conferencing"] or [mh "wireless technology"] or [mh internet] or [mh "information technology"] or [mh "mobile applications"]	12743
#6	(tele* or virtual* or video or remote* or "web based" or technolog* or online or "mobile app*" or software or phone or internet):ti,ab	137796
#7	(exercise* or rehabilitat* or physiotherap* or "physical therap*"):ti,ab	146032
#8	(physical* NEXT activ*):ti,ab	36900
#9	#7 or #8	166485
#10	#9 and #6	22937
#11	#10 or #5	33893
#12	[mh rehabilitation] or [mh exercise] or [mh "physical therapy modalities"] or [mh "physical therapy speciality"] or [mh "physical therapists"]	62360
#13	#12 or #9	191391
#14	#13 and #11 and #4 with Cochrane Library publication date from Jan 1998 to present	511

For peer review only

Websites:

The names of websites to be searched alongside search terms are provided below. On each website, authors will review the first three pages of search results. If there is no search function within a website, authors will look under relevant sections on that website, such as 'publications' or 'conferences'.

Websites to be searched:
MS Society, MS Trust, Chartered Society of Physiotherapy, UK Society of Behavioural Medicine, International Society of Behavioural Medicine, Open Grey, National MS Society, Rehabilitation in MS (RIMS), European Committee for Treatment and Research in MS.

Search terms:
Tele, virtual, multiple sclerosis, exercise
